# Isolation of four serotypes of epizootic hemorrhagic disease virus from *Culicoides* spp. and their associated infections in cattle in Yunnan, China

**DOI:** 10.1128/msphere.00274-25

**Published:** 2025-07-31

**Authors:** Zhenxing Yang, Yuwen He, Jinxin Meng, Susheng Li, Nan Li, Jinglin Wang, Jianling Song

**Affiliations:** 1 Yunnan Tropical and Subtropical Animal Viral Disease Laboratory, Key Laboratory of Transboundary Animal Diseases Prevention and Control (Co-construction by Ministry and Province), Yunnan Animal Science and Veterinary Institute 667624, Kunming, China; Instituto de Biotecnologia/UNAM, Cuernavaca, Morelos, Mexico

**Keywords:** epizootic hemorrhagic disease virus, *Culicoides *spp, virus isolation, complete genome analysis, cattle, China

## Abstract

Epizootic hemorrhagic disease (EHD) is a noncontagious viral infection transmitted by certain species of the genus *Culicoides* and has been included on the World Organization for Animal Health (OIE) list of diseases affecting domestic and wild ruminants. This study isolated four serotypes of EHDV strains (EHDV-1, -5, -6, and -7) from three *Culicoides* species collected in Yunnan Province, China. The phylogenetic analysis of Seg-3 revealed that EHDVs can be classified into two groups based on their geographical origins: Eastern and Western. The four isolates examined in this study belong to the Eastern group and are genetically most similar to EHDVs found in Japan and Australia. In the Seg-2 and Seg-6 phylogenetic trees, all EHDV serotypes can be classified into four clusters (Groups A–D). The four strains were categorized into Groups A, B, C, and D and belong to the EHDV-1, -5, -6, and -7 serotypes, respectively. Sero-epidemiological surveys were conducted on cattle in the border areas of China, Laos, Myanmar, and Vietnam using C-ELISA and neutralization tests. The rate of EHDV antibody positivity ranged from 29.22% to 58.86%, with an average of 43.09%. These surveys also revealed the presence of seven serotypes of EHDV circulating among cattle in these regions, resulting in diverse prevalence rates and spatial distributions. This finding indicates that EHDV circulates among natural vectors and infects local domestic cattle in these areas. The data gathered herein will help monitor the evolutionary characteristics of EHDV and its potential to cause outbreaks in China and neighboring countries.

**IMPORTANCE:**

EHDV is the causative agent of epizootic hemorrhagic disease (EHD), a viral illness with significant implications for animal welfare, society, and the economy. There are reports of EHDV-1, -6, -7, and -10 serotypes isolated from China, but no reports of EHDV-5 strains have been documented. Here, we report the isolation and identification of four serotypes of EHDV (EHDV-1, -5, -6, and -7) from three *Culicoides* species (*C. tainanus*, *C. oxystoma*, and *C. jacobsoni*) collected in Yunnan, China. Phylogenetic analysis revealed that the four strains of EHDV are most closely related to the Australian and Japanese strains. Sero-epidemiological survey results showed that in the border areas of China, Laos, Myanmar, and Vietnam, the rate of EHDV antibody positivity ranged from 29.22% to 58.86%, and seven serotypes of EHDVs circulating among cattle in these regions presented diverse prevalence rates and spatial distributions. This finding indicates that EHDV not only circulates among natural vectors but also infects local domestic cattle in these areas and that the EHDVs prevalent in the Asia-Pacific region may share a common gene pool.

## INTRODUCTION

Epizootic hemorrhagic disease virus (EHDV) is categorized under the genus *Orbivirus*, as part of the family *Reoviridae* (1). This arthropod-borne virus (arbovirus) is transmitted by biting midges of *Culicoides* spp. and infects domestic and wild ruminants, mainly white-tailed deer and cattle, causing epizootic hemorrhagic disease (EHD) (2, 3). EHD was first identified as a highly deadly disease of wild white-tailed deer in North America in 1955, and the virus was isolated from samples collected from animals affected by the disease (4). This disease can result in severe hemorrhagic syndrome in white-tailed deer, commonly leading to high mortality rates (3). EHD can also cause abortions or stillbirths, reduced milk production, fever, anorexia, and severe oral lesions in cattle. These effects may trigger costly investigations for foreign animal diseases on the affected premises, which also restrict the movement of livestock (2). Given its impact on deer and cattle, EHD has been on the World Organization for Animal Health (OIE) list of notifiable diseases since 2008 (5). This disease is widespread across the Americas, Africa, Europe, and Asia. However, various human activities, climate change, and increased world trade enhance the risk of disease emergence and worldwide distribution (6).

The EHDV genome, like those of other orbiviruses, consists of 10 distinct linear segments of double-stranded RNA (dsRNA) that encode seven structural proteins (VP1–VP7) and three nonstructural proteins (NS1–NS3/3a) (7, 8). The structural proteins VP2 and VP5, encoded by Seg-2 and Seg-6, are highly variable and form the outer capsid of the EHDV virion, which is correlated with the serotype (9). The core protein VP7, encoded by Seg-7, has a highly conserved amino acid sequence and expresses antigenic determinants common to all viruses in the EHDV serogroup (7). This protein is often utilized in serogroup-specific enzyme-linked immunosorbent assays (ELISAs) to diagnose viral infections (10). The subcore shell viral protein VP3, encoded by Seg-3, is highly conserved, displaying over 95.5% amino acid sequence identity among the EHDVs (7). Phylogenetic analyses of Seg-3 can classify EHDVs into different groups according to their geographic origins (11).

To date, seven serotypes of EHDV (designated EHDV-1, EHDV-2, and EHDV-4 to -8) and at least two new serotypes (EHDV-10 and another unnamed serotype) have been identified through extensive phylogenetic studies, sequencing data, and cross-neutralization assays (9, 11, 12). Genetic analyses revealed that the previously identified EHDV-3 (Nigerian strain Ib Ar 22619) is the serotype EHDV-1 (9). EHDV-2, traditionally known as the Ibaraki virus, caused an extensive disease outbreak in Japanese cattle in 1959, leading to 4,000 cattle deaths, and continues to cause sporadic disease outbreaks among cattle in the Far East (13, 14). In addition, infection outbreaks in Réunion Island and the United States have underscored the capacity of serotypes 6 and 7 to induce severe disease in cattle (15, 16). Notably, significant economic impacts have been observed due to EHDV-6 outbreaks in various locations, including Morocco (2004 and 2006), Algeria (2006), Turkey (2007), and Israel (2015) (17). Furthermore, an EHDV-7 outbreak in 2006 led to a loss of $2.5 million in the Israeli dairy cattle industry, mainly because of decreased milk production (18, 19). In the fall of 2021, a significant outbreak of EHDV-8 was reported on dairy and beef farms in Tunisia, presenting symptoms similar to those of bluetongue disease (20). The disease later spread to southern Italy, Spain, Portugal, and, more recently, France, resulting in severe cattle infections (21–23).

Although no outbreaks of EHD have been reported on the Chinese mainland, EHDV antibodies, including serotypes EHDV-1, -5, -6, -7, -8, and -10, have been detected in the serum of cattle and goats in southern Chinese provinces (23–25). To date, strains of EHDV-1, -7, and -10 have been isolated from sentinel cattle and *Culicoides* spp. in China, and a novel strain not belonging to any known serotype was recently isolated from cattle in Yunnan Province (12, 24, 26). While Chinese journals reported the isolation of EHDV-6 strains from sentinel cattle in Yunnan Province in 2012, no corresponding nucleotide sequences for these strains were published (27). Here, we report the complete genome sequences of EHDV serotypes 1, 5, 6, and 7 isolated from *Culicoides* spp. collected in Shizong, Jinghong, and Mangshi Counties, Yunnan Province, China. This is the first study in which the complete genome sequences of EHDV-5 and EHDV-6 strains have been reported in China. To our knowledge, only two EHDV-5 strains have previously been documented: one isolated in Australia in 1977 and another in Japan in 2016 (28). In addition, this report details the isolation, identification, and genomic characterization of these virus isolates. Furthermore, this study aimed to determine the seroprevalence of EHDV in cattle and characterize the spatial distribution of various EHDV serotypes in the border regions of China, Laos, Myanmar, and Vietnam.

## MATERIALS AND METHODS

### Collection and sorting of *Culicoides* specimens

**Fig 1:**
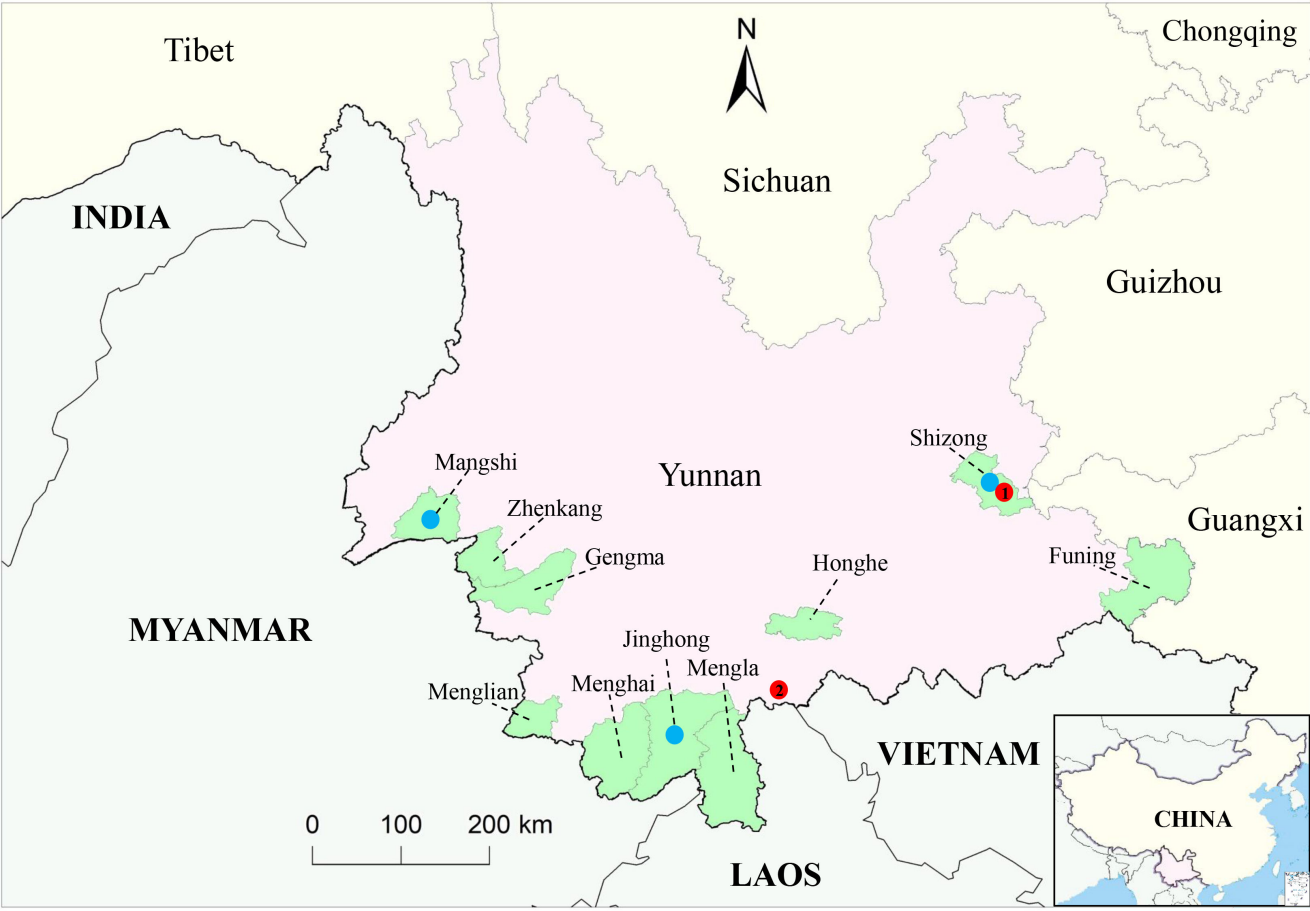
The geographical locations of *Culicoides* spp. and bovine serum sampling sites in Yunnan Province, China. Blue dots ● represent *Culicoides* spp. sampling sites, while green highlights the 10 counties from which bovine serum was collected. The red dots ● indicate the areas where EHDV strains have been isolated, with number 1 indicating the isolation of EHDV-1 and EHDV-7 from *Culicoides* spp. collected there in 2019 (26) and number 2 indicating the isolation of EHDV-10 from *Culicoides* (*C. tainanus*) collected there in 2013 (24). The maps were developed using the open-source GIS software QGIS 3.22, which utilized GPS coordinates and shapefiles from Natural Earth, a free GIS data source.

Between 2023 and 2024, midge specimens were collected from bovine shelters using light traps (12 V, 300 mA) from 6 p.m. to 8 a.m. the following day in the suburbs of Jinghong, Mangshi, and Shizong Counties in Yunnan Province (Fig. 1). The *Culicoides* species were preliminarily identified based on their gross morphology and wing patterns (29). Each hundred *Culicoides* spp. with similar morphologies collected from the same site were placed in a single cryopreservation tube, rapidly frozen in a liquid nitrogen tank, and transported to the laboratory for further analysis.

### Serum sample collection

In 2022, 3,460 blood samples were collected from beef cattle farms in 10 counties of Yunnan by local animal disease prevention and control centers. The number of samples collected from each county was as follows: Jinghong County (450 samples), Menghai County (425 samples), Mengla County (420 samples), Mangshi County (425 samples), Gengma County (160 samples), Zhenkang County (150 samples), Menglian County (350 samples), Funing County (450 samples), Honghe County (350 samples), and Shizong County (280 samples). The serum samples were separated from the blood samples through centrifugation, frozen at −20°C, and transported to the laboratory using a cold chain. To date, no EHDV vaccine has been used in China, and cattle have not been previously immunized with any EHDV vaccine.

### Cell cultures and viral isolation

BHK-21 (baby hamster kidney) cells were maintained at 37°C and 5% CO_2_ in 10% Dulbecco’s modified Eagle’s medium (DMEM) supplemented with 10% fetal bovine serum (FBS). C6/36 (*Aedes albopictus*) cells were maintained at 28°C and 5% CO_2_ in 50% DMEM plus 50% Rosewell Park Memorial Institute 1640 (RPMI) media supplemented with 10% FBS. All media were supplemented with 50 U/mL penicillin and 50 µg/mL streptomycin.

For virus isolation, as described in previous reports (30), each pool comprising 100 identical *Culicoides* of the same species was washed twice in 1 mL of PBS and then homogenized in 1 mL of ice-cold MEM containing 100 U/mL penicillin and 100 µg/mL streptomycin via the high-throughput tissue grinding instrument Scientz-48 (Ningbo Scientz Biotechnology Co., China). The homogenates were centrifuged at 10,000 × *g* for 2 min at 4°C, the supernatant was passed through a 0.22 micron filter unit (Merck Millipore Ltd. Co. Cork, Ireland), and 100 µL of the filtrate was inoculated onto monolayers of BHK-21 and C6/36 cells in 24-well culture plates. The cells were monitored daily for cytopathic effects (CPEs) across three successive passages, and supernatants from cultures exhibiting CPEs were stored at −80°C for further identification.

### Extraction of viral dsRNA and initial virus identification

Total RNA was extracted from virus-infected cells using RNAiso Plus (TaKaRa). The viral dsRNA was then separated from the total RNA by precipitating high-molecular-mass-weight single-stranded RNA (ssRNA) in 2 M LiCl (31). As previously reported (26, 32), the extracted RNA was denatured at 95°C, rapidly cooled on ice, and tested via RT-qPCR with primers and probes for EHDV, Akabane virus (AKAV), Banna virus (BAV), Bluetongue virus (BTV), Tibet Orbivirus (TIBOV), and Palyam virus (PALV). RT-qPCR was conducted on a 7500 Fast Real-Time PCR machine (Applied Biosystems, Carlsbad, CA, USA) under the following conditions: 45°C for 5 min, 95°C for 10 s, followed by 40 cycles of 95°C for 5 s and 60°C for 34 s.

### Full-length cDNA amplification and next-generation sequencing

The genomic segments of the four isolates were reverse-transcribed into cDNA using the full-length amplification of cDNAs (FLAC) technique (33, 34). An anchor primer with a phosphorylated 5′ terminus was ligated to the 3′ ends of the viral dsRNA using T4 RNA ligase I (NEB, USA) at 37°C overnight. After purification with the Monarch PCR & DNA Cleanup Kit (NEB, USA), reverse transcription was carried out using PrimeScript II Reverse Transcriptase (TaKaRa). The resulting cDNAs were amplified using PrimeSTAR GXL DNA Polymerase (TaKaRa) and a 1-15-1 primer complementary to the anchor primer via the following procedure: 94°C for 5 min, followed by 30 cycles of 94°C for 30 s, 55°C for 30 s, and 68°C for 4 min.

The amplified products were analyzed on a 1% agarose gel (90 V for 2–3 h) using Goldview II dye (Solarbio, China) and subsequently sent to Magigen Company (Guangzhou, China) for next-generation sequencing (NGS) on the Illumina HiSeq 2000 platform (Illumina, San Diego, CA, USA). Several filtering steps were employed to eliminate low-quality sequences, including those originating from PCR or sequencing adapters, ensuring data quality for *de novo* assembly. Next, *de novo* assembly was conducted using AbySS v2.0.2 (35) and SOAPdenovo (36) to edit and assemble the genomic segments of the isolated virus. Based on the sequencing results of NGS, multiple pairs of primers were designed for RT-PCR and Sanger sequencing. The two sequencing results were then compared, and the verified sequences were submitted to GenBank, and the accession numbers are listed in Table 2.

### Sequence analysis and phylogenetic tree construction

The reference sequences of EHDVs and an outgroup (BTV) were downloaded from GenBank on January 31, 2025, and are presented in Table S1 (Supporting Information). The open reading frames (ORFs) of viral genomic segments were identified and translated into amino acid sequences using ORFfinder (https://www.ncbi.nlm.nih.gov/orffinder/). Multiple alignments of the consensus sequences were conducted using MAFFT v7.520 (37), and the nucleotide/amino acid identities were calculated using BioAider v1.627 (38). Pairwise distance calculations and phylogenetic tree constructions were conducted using MEGA-11 (https://www.megasoftware.net) with the neighbor-joining (NJ) method, pairwise deletion, and the maximum composite likelihood algorithm, and were tested by bootstrapping with 1,000 replicates.

### Serological analyses

First, the serum samples were analyzed using a competitive ELISA (C-ELISA) kit (23) developed in our laboratory to detect EHDV group-specific antibodies. From these samples, 100 positive serum samples were randomly selected from each region to assess the presence of serotype-specific EHDV (EHDV-1,-2, -5,-6, -7,-8, and −10) antibodies using a serum neutralization test (SNT). EHDV-1, -5, -6, and -7 strains are reported in this study, whereas the EHDV-10 strain (24) was previously isolated and preserved in our laboratory. The EHDV-2 and -8 strains originated from the Elizabeth Macarthur Agricultural Institute.

SNT was conducted according to previously reported methods (39, 40). The TCID_50_/50 µL of each different serotype of EHDV was determined by titrating 10-fold dilutions of the supernatant on BHK-21 cells in 96-well flat-bottomed microtiter plates. Serum samples designated for testing were heat-inactivated at 56°C for 30 min before being diluted (1:10–1:1280) in a tissue culture medium free of FBS. Each dilution was mixed with an equal volume of virus containing 100 TCID_50_/50 µL and incubated for 1 hour at 37°C with 5% CO_2_. After this incubation, 100 µL of a BHK-21 cell suspension (1 × 10^5^ cells/mL) was added to each well and subsequently incubated for 5–7 days at 37°C; then, the wells were assessed for CPEs. The neutralization titer was determined as the serum dilution that produced the 50% neutralization endpoint. It was calculated via the Spearman–Kaerber method and expressed as the log^10^ reciprocal of the highest positive plasma dilution. The serum is deemed positive for that serotype when the neutralizing serum titer for a specific EHDV serotype is greater than or equal to 1:16.

## RESULTS

### *Culicoides* collection

Between 2023 and 2024, we collected samples of *Culicoides* spp. from bovine shelters in the suburbs of Jinghong, Mangshi, and Shizong Counties in Yunnan Province. A total of 39,000 samples were collected at these three sites, encompassing nine distinct species (*C. tainanus*, *C. jacobsoni*, *C. sumatrae*, *C. oxystoma*, *C. kinabaluensis*, *C. insignipennis*, *C. actoni*, and C. arakawai), and *C. trithecoides* samples belonging to this subgenus had yellow scutum (Table 1). However, the predominant species composition varies from location to location. Notably, *C. tainanus* (comprising 25.63%) and *C. sumatrae* (constituting 21.88%) emerged as the predominant species among the collected *Culicoides* spp. in Jinghong, and these two species were also the predominant species in Shizong, accounting for 29.17% and 30.83%, respectively. The predominant species of *Culicoides* spp. collected in Mangshi were *C. tainanus* (23.64%) and *C. oxystoma* (29.09%), but in general, *C. tainanus* was the predominant species at all three sampling sites. To facilitate our research, the 39,000 *Culicoides* samples were carefully divided into 390 pools, categorized by the eight morphologically identified species and the mixed species of *Culicoides* belonging to the subgenus *Trithecoides* that could not be identified. These pools were homogenized and inoculated with BHK-21 and C6/36 cells to isolate viruses.

**TABLE 1:** *Culicoides* biting midges collected from the suburbs of Jinghong, Mangshi, and Shizong counties in Yunnan Province, China

Geographic origin (County)	Longitude and latitude	Altitude (m)	Collection date	Number and proportion (%) of different species									Total
				*C. tainanus*	*C. jacobsoni*	*C. sumatrae*	*C. oxystoma*	*C. kinabaluensis*	*C. insignipennis*	*C. actoni*	*C. arakawai*	*C. trithecoides* ^*a*^	
Jinghong	100°51′32″ E, 22°5′16″ N	814	October 15, 2024	4,100 25.63%	2,300 14.38%	3,500 21.88%	1,300 8.13%	1,700 10.63%	1,100 6.88%	100 0.63%	500 3.13%	1,400 8.75%	16,000
Mangshi	98°30'30″ E, 24°21'22″ N	884	June 15, 2023	2,600 23.64%	1,100 10%	1,000 9.09%	3,200 29.09%	200 1.82%	600 5.45%	200 1.82%	800 7.27%	1,300 11.82%	11,000
Shizong	104°17′28″ E, 24°38′53″ N	974	July 17, 2023	3,500 29.17%	1,600 13.33%	3,700 30.83%	1,000 8.33%	400 3.33%	500 4.17%	100 0.83%	300 2.50%	900 7.50%	12,000
Total				10,200 26.15%	5,000 12.82%	8,200 21.03%	5,500 14.10%	2,300 5.90%	2,200 5.64%	400 1.03%	1,600 4.10%	3,600 9.23%	39,000

^*a*^ *C. trithecoides* refers to specimens within this subgenus with a yellow scutum.

### Virus isolation and primary identification

After the homogenized suspension was inoculated onto C6/36 cells and passaged three times in BHK-21 cells, eight isolates that caused strong CPEs on BHK cells at 48 hours post-inoculation were characterized by cell rounding, lysis, and shedding. However, two additional isolates were unable to cause CPEs on BHK cells. Still, they could cause CPEs on C6/36 cells. RNA samples were extracted from the supernatants of cells infected with viral isolates and identified via RT-qPCR screening for the viral nucleotide sequences of BTV, EHDV, PALV, AKAV, TIOBV, and BAV. Consequently, four strains of EHDV, three strains of BTV, one strain of PALV, and two strains of BAV (BAV does not cause CPEs on BHK cells) were identified. Then, EHDV serotypes were analyzed using serotype-specific RT-qPCR with eight pairs of primers and probes (Table S2) (41), the results indicated that the four EHDV strains (JH24C022, SZ23C107, MS23C056, and JH24C130) corresponded to four distinct serotypes: EHDV-1, -5, -6, and -7, respectively. EHDV-1 and EHDV-6 were isolated from *C. tainanus*, EHDV-7 was isolated from *C. oxystoma*, and EHDV-5 was isolated from *C. jacobsoni* (Table 2).

**TABLE 2:** Isolation details of four viruses isolated from *Culicoides* spp. collected in Jinghong, Mangshi, and Shizong counties in Yunnan Province, China

Isolates^*a*^	Identification	Location	Hosts	GenBank accession no.
JH24C022	EHDV-1	Jinghong	*C. tainanus*	PV476106–PV476115
JH24C130	EHDV-7	Jinghong	*C. oxystoma*	PV476116–PV476125
MS23C056	EHDV-6	Mangshi	*C. tainanus*	PV476126–PV476135
SZ23C107	EHDV-5	Shizong	*C. jacobsoni*	PV476136–PV476145

^*a*^ JH, MS, and SZ refer to the location of the midge collection site, Jiangcheng, Mangshi, and Shizong; the numbers 23 and 24 refer to the years 2023 and 2024, respectively, which indicate the years in which the samples were collected; C refers to the *Culicoides* spp.; the number following the letter "C" indicates the midge pool numbers.

### NGS sequencing and genomic characteristics of the isolated virus

The genomic dsRNAs of the four EHDV strains were amplified using the FLAC technique and subjected to genome sequencing on the Illumina HiSeq 2000 platform. Complete genome sequences for Seg-1 to Seg-10 of the four isolates were obtained and submitted to GenBank under the accession numbers PV476106–PV476115 for JH24C022, PV476116–PV476125 for JH24C130, PV476126–PV476135 for MS23C056, and PV476136–PV476145 for SZ23C107 (Table 2). Detailed information regarding each gene fragment of each virus, along with the corresponding length of the encoded protein and their respective gene numbers, is found in Table S3.

The genome lengths of the four EHDV strains ranged from 19,300 bp to 19,364 bp, with G + C contents varying between 41.93% and 42.42%. Their 10 segments contained variable numbers of nucleotides (nt), ranging from 3,942 bp for Seg-1 to 810 bp for Seg-10, encoding proteins with differing amino acid residues (aa) from 1,302 for VP1 to 228 for NS3. The six nucleotides at the 5′ end and the five nucleotides at the 3′ end of all 10 segments demonstrated a high degree of conservation, appearing as 5′-GUUAAA and CUUAC-3′. Like most orbiviruses, the 5′ noncoding regions (NCRs) were shorter than the 3′ NCRs, and the first and last two nucleotides of each segment are reverse complements (Table S3). These four strains, 5′ and 3′ NCRs, accounted for 3.77% to 3.86% of their respective total genomes.

### Nucleotide and amino acid identity analysis of the isolated virus

The sequences of the four EHDV isolates were compared with those of other EHDVs in GenBank, and the results are shown in Table 3. The JH24C022 strain presented the highest homology with the Chinese EHDV-1 strain YNV/KM3 in all segments except Seg-8. The identity levels ranged from 95.1% to 98.5% at the nucleotide level (nt) and 97.6% to 100% at the amino acid level (aa). By contrast, Seg-8 showed lower identity, with identities of only 70.4% (nt) and 69.2% (aa). However, the identity of Seg-8 (NS2) between JH24C022 and the Japanese EHDV-1 strain Kawanabe_525 was as high as 97.3% (nt) and 97.1% (aa), respectively. MS23C056 strain had the closest relationship with the Japanese EHDV-6 strain ON-3/E/14, with nt and aa identity levels of 98.1–98.9% and 94.6–100%, respectively. JH24C130 strain was more closely related to the Japanese EHDV-7 strain FO-1/E/16, with nt and aa identities of 98.9–99.3% and 98.5–100%, respectively. The most interesting finding was that the SZ23C107 strain is closely related to eight EHDVs (serotypes 1, 5, 7, 8, and 10) isolated from Australia, Japan, and China in each genome segment. The identity among the different segments is 92.8%–99.6% (nt) and 95.9%–100% (aa), respectively.

**TABLE 3:** Summary of the EHDV strains with the highest sequence identity to the four isolates in this study

Genome segment	EHDV strains most closely related to the four isolations in the study	GenBank accession no.	Sequence identity (%)	
			Nucleotide	Amino acid
EHDV-1_JH24C022				
Seg-1 (VP1)	EHDV-1_YNV/KM3_China	OM953791	98.5	99.5
Seg-2 (VP2)	EHDV-1_YNV/KM3_China	OM953792	96.2	97.6
Seg-3 (VP3)	EHDV-1_YNV/KM3_China	LC202971	97.8	99.6
Seg-4 (VP4)	EHDV-1_YNV/KM3_China	OM953794	98.0	99.5
Seg-5 (NS1)	EHDV-1_YNV/KM3_China	OM953795	98.3	99.2
Seg-6 (VP5)	EHDV-1_YNV/KM3_China	LC202960	98.4	100.0
Seg-7 (VP7)	EHDV-1_YNV/KM3_China	OM953797	96.9	100.0
Seg-8 (NS2)**^*a*^**	EHDV-1_Kawanabe_525_Japan	LC552738	97.3	97.1
Seg-9 (VP6)	EHDV-1_YNV/KM3_China	OM953799	98.5	97.6
Seg-10 (NS3)	EHDV-1_YNV/KM3_China	OM953800	95.1	100.0
EHDV-5_SZ23C107				
Seg-1 (VP1)	EHDV-8_AUS1982/06_CPR_3961A_Australia	AM745057	95.9	98.8
Seg-2 (VP2)	EHDV-5_ON-11/E/16_Japan	LC757714	92.8	95.9
Seg-3 (VP3)	EHDV-7_YN09-04_China	MK656455	98.0	99.8
Seg-4 (VP4)	EHDV-10_ON-4/B/98_Japan	LC552749	98.9	99.3
Seg-5 (NS1)	EHDV-10_JC13C644_China	MT013328	98.9	99.6
Seg-6 (VP5)	EHDV-5_AUS1977/01_CSIRO_157_Australia	AM745032	93.7	99.2
Seg-7 (VP7)	EHDV-5_AUS1977/01_CSIRO_157_Australia	AM745033	94.3	99.7
Seg-8 (NS2)	EHDV-1_YNV/KM3_China	OM953798	96.4	96.2
Seg-9 (VP6)	EHDV-10_JC13C673_China	MT013322	99.4	99.4
Seg-10 (NS3)	EHDV-10_JC13C673_China	MT013323	99.6	100.0
EHDV-6_MS23C056				
Seg-1 (VP1)	EHDV-6_ON-3/E/14_Japan	LC757723	98.1	99.6
Seg-2 (VP2)	EHDV-6_ON-3/E/14_Japan	LC757724	98.3	98.9
Seg-3 (VP3)	EHDV-6_ON-3/E/14_Japan	LC757725	98.1	99.6
Seg-4 (VP4)	EHDV-6_ON-3/E/14_Japan	LC757726	98.5	99.6
Seg-5 (NS1)	EHDV-6_ON-3/E/14_Japan	LC757727	98.9	99.0
Seg-6 (VP5)	EHDV-6_ON-3/E/14_Japan	LC757728	98.1	99.8
Seg-7 (VP7)	EHDV-6_ON-3/E/14_Japan	LC757729	98.7	100.0
Seg-8 (NS2)	EHDV-6_ON-3/E/14_Japan	LC757730	98.2	98.1
Seg-9 (VP6)	EHDV-6_ON-3/E/14_Japan	LC757731	98.1	94.6
Seg-10 (NS3)	EHDV-6_ON-3/E/14_Japan	LC757732	98.7	100.0
EHDV-7_JH24C130				
Seg-1 (VP1)	EHDV-7_FO-1/E/16_Japan	LC599904	99.1	99.6
Seg-2 (VP2)	EHDV-7_FO-1/E/16_Japan	LC552731	99.2	99.3
Seg-3 (VP3)	EHDV-7_FO-1/E/16_Japan	LC552732	99.2	99.8
Seg-4 (VP4)	EHDV-7_FO-1/E/16_Japan	LC599905	99.2	99.8
Seg-5 (NS1)	EHDV-7_FO-1/E/16_Japan	LC599906	99.1	99.8
Seg-6 (VP5)	EHDV-7_FO-1/E/16_Japan	LC552733	99.3	99.8
Seg-7 (VP7)	EHDV-7_FO-1/E/16_Japan	LC599907	99.2	100.0
Seg-8 (NS2)	EHDV-7_FO-1/E/16_Japan	LC599908	99.2	99.2
Seg-9 (VP6)	EHDV-7_FO-1/E/16_Japan	LC599909	98.9	98.5
Seg-10 (NS3)	EHDV-7_FO-1/E/16_Japan	LC599910	99.0	99.5

^*a*^ The identity of Seg-8 (NS2) between JH24C022 and YNV/KM3 is only 70.4% and 69.2% at the nt and aa levels, respectively.

### Phylogenetic analysis of the four EHDV strains

**Fig 2:**
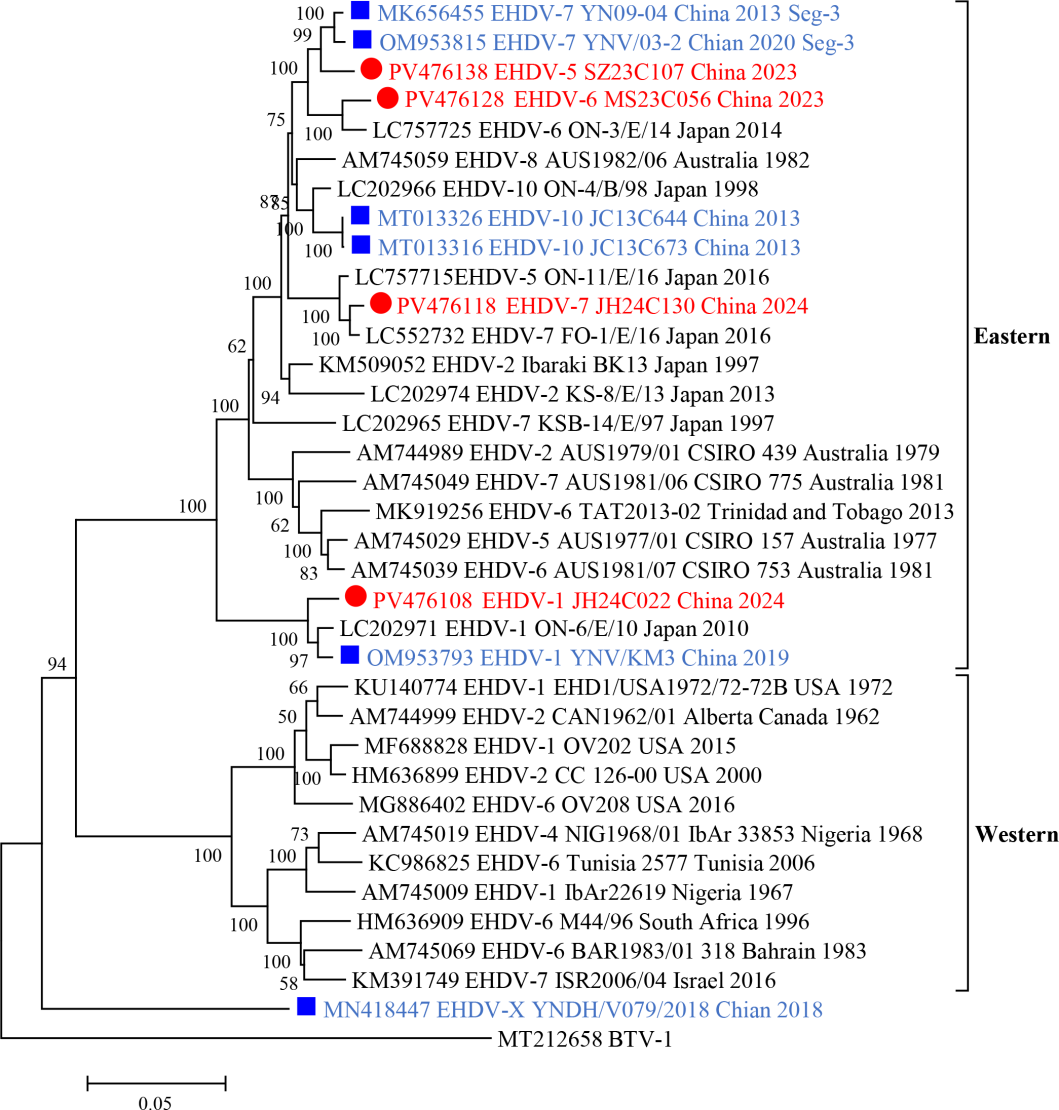
Phylogenetic analysis based on the coding sequences of Seg-3 of the four isolates in the study with reference strains of recognized EHDV. Each reference EHDV strain is denoted as “GenBank accession number_ Serotype_ Strains number_ Country_ Date.” Outgroup viruses are denoted as “GenBank accession number_ Virus name.” Red dots represent the isolates in this study, while blue squares denote other EHDV strains isolated in China.

Phylogenetic analyses of genomic segments revealed that EHDVs can be divided into two groups, known as “Eastern” or “Western,” according to their geographic origins (7, 42). Phylogenetic analysis of the complete coding sequence of Seg-3, including Chinese strains and other strains isolated from different regions, revealed that all the Chinese strains clustered into an eastern group alongside the Japanese and Australian strains (Fig. 2). However, a Chinese strain, YNDH/V079/2018, did not cluster with the identified Eastern or Western groups, forming a distinct branch on the phylogenetic tree of Seg-3. The identities of Seg-3/VP3 nt and aa between the four EHDV strains and the Eastern and Western strains were 90.4%–99.2%/98.6%–99.8% (nt/aa) and 79.8%–92.2%/95.6%–99.5% (nt/aa), respectively (Table S4). These findings indicate that the four strains isolated from *Culicoides* spp. in Yunnan Province are part of the Eastern EHDV group. Similar results were also observed in the phylogenetic tree analysis of five additional gene segments: Seg-1, Seg-4, Seg-5, Seg-8, and Seg-9 (Fig. S1 to S5). Interestingly, the JH24C022 strain was clustered into an independent branch with the Kawanabe_525 strain in the phylogenetic tree of Seg-8; however, it was clustered together with the YNV/KM3 strain in the phylogenetic trees of other genomic segments (Fig. S1 to S5).

**Fig 3:**
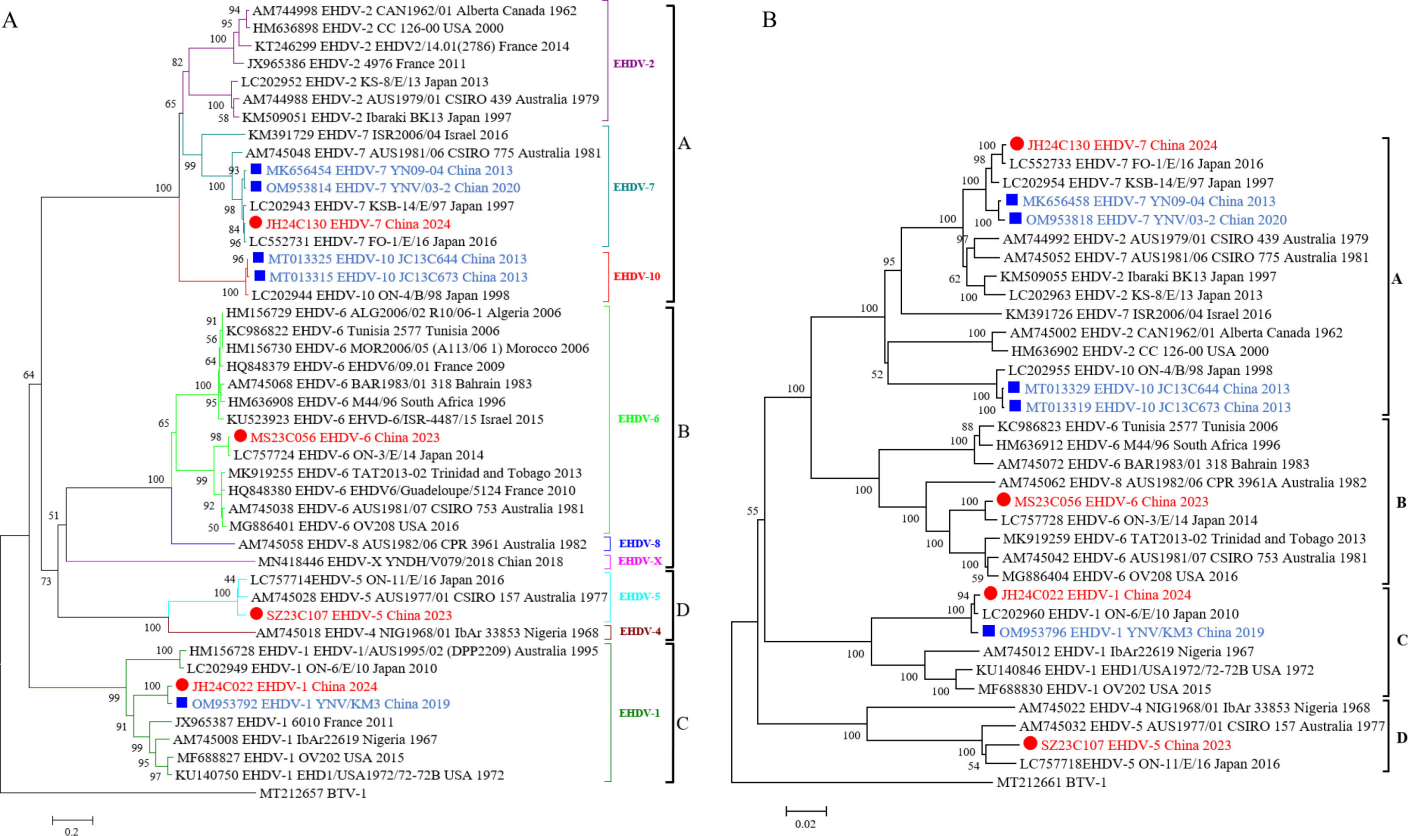
Phylogenetic analysis based on the coding sequences of Seg-2 (**A**) and Seg-6 (**B**) of the four isolates in the study with reference strains of recognized EHDV. Each reference EHDV strain is denoted as “GenBank accession number_ Serotype_ Strains number_ Country_ Date.” Outgroup viruses are denoted as “GenBank accession number_ Virus name.” Red dots represent the isolates in this study, while blue squares denote other EHDV strains isolated in China.

The outer capsid proteins VP2 and VP5 of EHDV, encoded by Seg-2 and Seg-6, exhibit high variability and are crucial for serotype determination. In the phylogenetic tree constructed for the complete coding sequences of Seg-2 and Seg-6, all EHDV serotypes could be grouped into four distinct clusters (Groups A–D), and the four EHDV strains in this study were sorted into Groups A, B, C, and D, respectively (Fig. 3). In Group A, JH24C130 was closely related to EHDV-7 strains (FO-1/E/16, KSB-14/E/97, YNV/03-2, and YN09-04) and shared 95.9%–99.2% (nt)/97.6%–99.3% (aa) identities for Seg-2/VP2 and 96.0%–99.3% (nt)/99.0%–99.8% (aa) identities for Seg-6/VP5 with these strains. In Group B, the MS23C056 strain was closely related to the EHDV-6 strains, sharing nt/aa identities of 71.5%–98.3%/75.5%–98.9% for Seg-2/VP2 and 79.9%–98.1%/96.3%–99.8% for Seg-6/VP5. In Group C, the JH24C022 strain was closely related to the EHDV-1 strains, with identities ranging from 71.1% to 96.2%/74.0%–97.6% (nt/aa) for Seg-2/VP2 and from 79.7%–98.4%/95.2%–100% (nt/aa) for Seg-6/VP5. In Group D, SZ23C107 was closely related to two strains of EHDV-5 (AUS1977/01 and ON-11/E/16) within a distinct evolutionary cluster, sharing nt/aa identities of 92.5%–92.8%/94.4%–95.9% for Seg-2/VP2 and 92.6%–93.7%/98.6%–99.2% for Seg-6/VP5 (Tables S5 and S6).

### Epidemiological investigation of EHDV in livestock

To further assess the dispersal of EHDV in Yunnan Province, we randomly selected 3,460 cattle serum samples from 10 counties in the region, including three areas where EHDV was isolated (Fig. 1), and screened them using C-ELISA. A total of 1,491 samples tested positive for antibodies against EHDV, resulting in an overall seropositive rate of 43.09% (95% CI, 41.44%–44.74%) (Table 4). The EHDV seropositivity rate was highest in Menglian at 58.86% (95% CI, 53.7%–64.01%) and lowest in Funing at 29.33% (95% CI, 25.13%–33.54%). Overall, the four counties (Menglian, Jinghong, Menghai, and Mengla), which are geographically close and located in the southern section of the Tropic of Cancer, had the highest EHDV antibody-positive rates, ranging from 45.95% to 58.86%.

**TABLE 4:** Results of antibody detection against EHDV using C-ELISA

Location	Sample numbers	Number of positive samples	Seroprevalence %	95% CI of seroprevalence %	
Jinghong	450	221	49.11	44.49	53.73
Menghai	425	202	47.53	42.78	52.28
Mengla	420	193	45.95	41.19	50.72
Mangshi	425	188	44.24	39.51	48.96
Gengma	160	59	36.88	29.40	44.35
Zhenkang	150	65	43.33	35.40	51.26
Menglian	350	206	58.86	53.70	64.01
Funing	450	132	29.33	25.13	33.54
Honghe	350	111	31.71	26.84	36.59
Shizong	280	114	40.71	34.96	46.47
Overall	3,460	1,491	43.09	41.44	44.74

**Fig 4:**
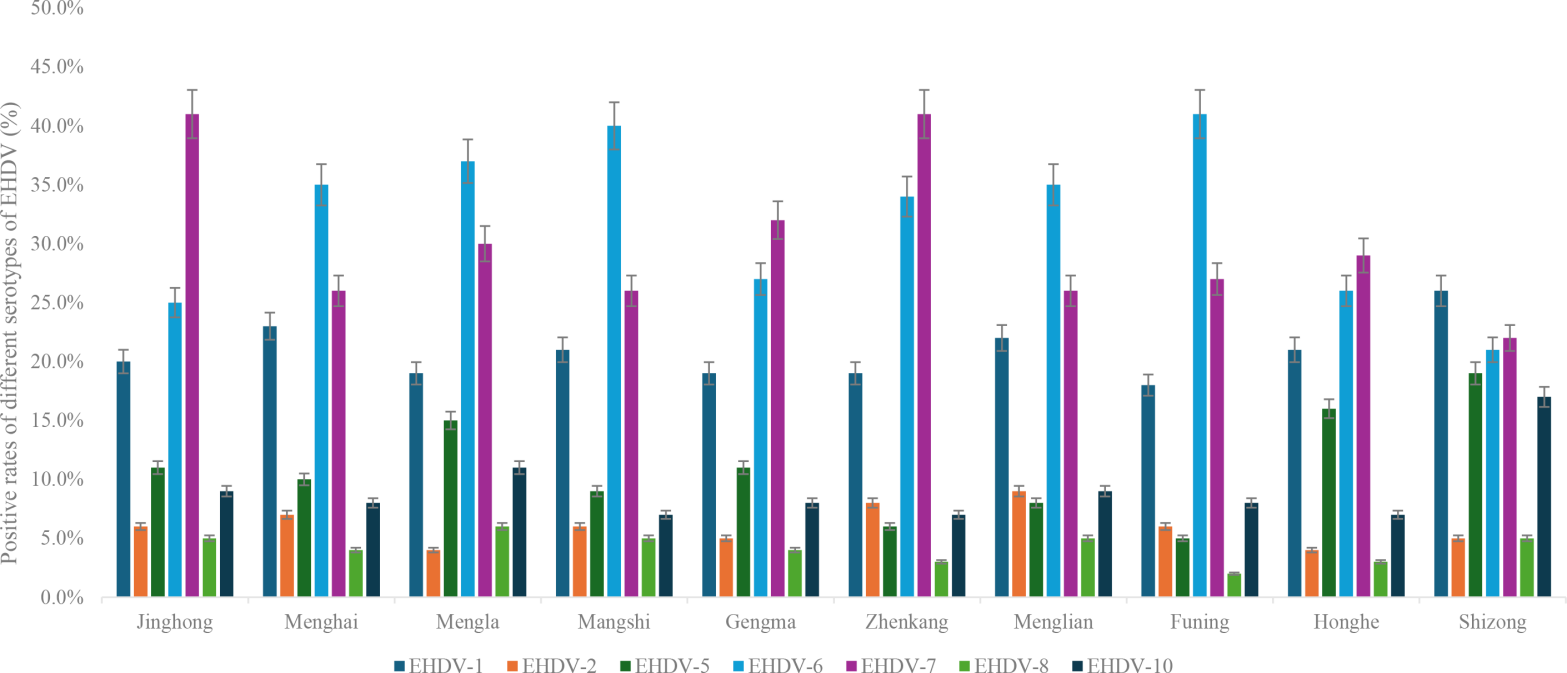
The detection of seven EHDV serotypes neutralizing antibodies in cattle across 10 counties in Yunnan Province, China.

One hundred positive serum samples from each region were randomly selected and titrated for antibodies specific to seven serotypes of EHDV (EHDV-1, -2, -5, -6, -7, -8, and -10). Antibodies against all seven EHDV serotypes were detected across the 10 regions. Among the detected serotypes, EHDV-6 had the highest seropositive rate at 32.1% (95% CI, 29.21%–34.99%), followed by EHDV-7, EHDV-1, EHDV-5, EHDV-10, EHDV-2, and EHDV-8 (Table S7). However, the distribution of EHDV serotypes varied by region (Fig. 4). Jinghong, Gengma, Zhenkang, and Honghe had the highest proportions of EHDV-7 serotypes. By contrast, Menghai, Mengla, Mangshi, Menglian, and Funing presented the highest proportions of EHDV-6 serotypes. Shizong had the highest proportion of EHDV-1 serotypes. In addition, the proportions of EHDV-10 and EHDV-5 in Shizong were greater than those in other regions, whereas the proportions of serotypes EHDV-2 and EHDV-8 were the lowest across all regions.

## DISCUSSION

*Culicoides* spp. are vectors of several arboviruses that are highly pathogenic to livestock, wildlife, and humans (43). Over 1,400 species of *Culicoides* spp. are found worldwide, with 168 species identified in Southeast Asia and more than 70 species in Yunnan Province, China, which borders Southeast Asia (44). However, only over 40 species are recognized as vectors or potential vectors for at least 50 arbovirus species, including BTV, EHDV, PALV, bovine ephemeral fever virus (BEFV), and African horse sickness virus (AHSV) (45). Our research group isolated or detected various arboviruses from *Culicoides* spp. collected in Yunnan Province during previous investigations, including BTV (46), EHDV (serotypes 1, 7, and 10) (24, 26), BAV (47), TIBOV (48), AKAV (49), and Oya virus (40). In this study, four EHDV strains were isolated from *Culicoides* spp. collected in this region.

The species composition of *Culicoides* spp. at the three sampling sites was different. The differences in the species composition and abundance of *Culicoides* spp. may vary based on the sampling environment and time. However, the species *C. tainanus* (comprising 26.15%) was predominant at all three sampling sites. If the species *C. jacobsoni*, *C. sumatrae*, and *C. oxystoma* are included, they represent 74.1% of the total collection. These four species of *Culicoides* spp. are widely distributed in China, Japan, Australia, and Southeast Asia, but only *C. tainanus*, *C. jacobsoni*, and *C. oxystoma* are considered potentially important vectors of EHDV or BTV (50, 51). There is currently no evidence that *C. sumatran* is associated with BTV or EHDV (44). Consistent with previous reports, the four EHDV strains reported in this study were also isolated from *C. tainanus*, *C. jacobsoni*, and *C. oxystoma* collected from bovine shelters in the suburbs of Jinghong, Mangshi, and Shizong Counties. This finding reaffirms that these three species of *Culicoides* spp. may act as potential vectors for local EHDV transmission. In addition, among the collected *Culicoides* spp., these three species represented 48.13% in Jinghong, 62.73% in Mangshi, and 50.83% in Shizong at each sampling point. This discovery highlights the crucial role that three species of *Culicoides* may play in the natural circulation of local EHDV, making continuous monitoring of their population distribution and density crucial. Indeed, our study has some limitations. The next step is to conduct continuous sampling of *Culicoides* spp. in the three regions to understand further the distribution of *Culicoides* density and species across different seasons.

The global distribution of EHDV serotypes shows distinct regional patterns. EHDV-1, -2, and -6 are prevalent in North America (52). EHDV-1,- 2, -3, -4, and -6 are found in Africa (6). Australia has reported EHDV-1, -2, -5, and -8 (53). EHDV-6 and EHDV-8 are epidemic in Europe (21, 22). The prevalent EHDV serotypes in Asia are diverse and include EHDV-1, -2, -6, -7, -8, and -10 (24, 28). Currently, EHDV-1, -6, -7, and -10 have been detected in China in cattle or *Culicoides* spp., but only the EHDV-1, -7, and -10 sequences are available. In this study, we first used serotype RT-qPCR (41) to detect the serotypes of four EHDV strains, and the results revealed that their serotypes were EHDV-1, -5, -6, and -7. We subsequently conducted genome sequencing and phylogenetic analysis of these four EHDV strains. The complete genome sequences of the four EHDV strains ranged from 19,300 bp to 19,364 bp, with G + C contents ranging from 41.93% to 42.42%. Like other EHDVs, six nucleotides at the 5′ end and five nucleotides at the 3′ end of all ten segments showed a high degree of conservation (5′-GUUAAA and CUUAC-3′). Sequence analysis revealed that the four isolates shared the highest identity with EHDV isolates from Australia, Japan, and China, with nucleotide and amino acid identities ranging from 92.8% to 99.6% (nt) and 94.6% to 100% (aa), respectively (Table 3).

Seg-3 encodes the highly conserved inner core protein VP3 of EHDV, which has over 95.5% amino acid sequence identity when all EHDV strains are considered (7). Phylogenetic analyses of Seg-3 coding sequences can categorize EHDV strains into Eastern and Western topotypes (42). The phylogenetic analysis of the Seg-3 coding sequences revealed that the four isolates clustered with all the Japanese, Australian, and Chinese strains within the eastern group. This relationship is emphasized by their high nt and aa identities, which range from 90.4% to 99.2% (nt) and 98.6% to 99.8% (aa), respectively. Furthermore, similar results were found in the phylogenetic tree of five other gene segments, Seg-1, Seg-4, Seg-5, Seg-8, and Seg-9 (Fig. S1 to S5), indicating that the Chinese strains are among the EHDVs found in the Asia-Pacific region. However, there is an exception: a strain of EHDV (YNDH/V079/2018) was isolated in China in 2018. The Seg-3 phylogenetic tree did not cluster with either the Eastern or Western strains but formed an independent branch, showing only 80.0% and 78.9% sequence identity with other EHDVs at the nt and aa levels, respectively (12). This finding indicates a potentially greater diversity of EHDVs in the Asia-Pacific region.

The outer capsid of EHDV comprises the proteins VP2 and VP5, which Seg-2 and Seg-6 encode. These proteins exhibit high variability and are closely related to viral serotypes (9). Therefore, phylogenetic analysis of these two proteins’ nt or aa sequences can identify serotypes of newly isolated strains. This study categorizes all EHDV strains into four distinct groups: A, B, C, and D, based on the phylogenetic analysis of the nt sequences of VP2 and VP5. In both phylogenetic trees, the four EHDV strains were clustered into Groups A, B, C, and D. The JH24C130 strain in Group A was identified as EHDV-7 by phylogenetic analysis of its VP2 nt sequence with other EHDV-7 strains (Fig. 3), and shared nt and aa identities of 74.5%–99.2%/79.6%–99.3% for Seg-2/VP2 and 81.5%–99.3%/97.3%–99.8% for Seg-6/VP5, respectively (Tables S5 and S6). The MS23C056 strain in Group B was identified as EHDV-6 through phylogenetic analysis of the VP2 nt sequences and shared nt and aa identities of 71.5%–98.3%/75.5%–98.9% for Seg-2/VP2 and 79.9%–98.1%/96.3%–99.8% for Seg-5/VP6 with EHDV-6 strains isolated from other locations (Tables S5 and S6). Notably, all segments of these two strains have the highest identity with the Japanese EHDV-7 strain FO-1/E/16 and the EHDV-6 strain ON-3/E/14, indicating that these EHDV strains isolated in China and Japan may have a common origin. Interestingly, high nt sequence identity was discovered among several other arboviruses, including BTV (54), PALV (55), and other different serotypes of EHDV (24, 56), which were isolated in the Asia-Pacific region, providing further evidence that arboviruses in this region may share the same gene pool.

The JH24C022 strain in Group C was identified as EHDV-1 by the phylogenetic analysis of its VP2 nt sequence with other EHDV-2 strains (Fig. 3), and they shared nt and aa identities of 71.1%–96.2%/74.0%–97.6% for Seg-2/VP2 and 79.7%–98.4%/95.2%–100% for Seg-6/VP5, respectively (Tables S5 and S6). Importantly, except for Seg-8, all segments of the JH24C022 strain had the highest identity with the Chinese YNV/KM3 strain, with 95.1%–98.5% (nt) and 97.6%–100% (aa) identity. In comparison, the identities of Seg-8 nt and aa were only 70.4% and 69.2%, respectively. However, Seg-8 of JH24C022 presented the highest identity with the Japanese strain Kawanabe_525, shared 97.3% and 97.1% identities at the nt and aa levels, respectively (Table 3). Similar results can also be seen in the phylogenetic tree. In the Seg-8 phylogenetic tree, JH24C022 and Kawanabe525 are clustered in one branch, whereas in the phylogenetic tree of the other segments, JH24C022 is clustered together with YNV/KM3. The JH24C022 strain may have originated from the Chinese EHDV-1 strain YNV/KM3, which was isolated in 2019; however, its Seg-8 may have been derived from the Japanese EHDV-1 strain Kawanabe525, suggesting reassortment between the two strains. The prevalence of Kawanabe525 in China is still unclear, making it impossible to determine whether the reassortment event occurred in China. Nevertheless, it can be confirmed that these two strains may have co-circulated in the Asia-Pacific region. Genetic reassortment likely played a crucial role in the evolution of orbiviruses, resulting in genotypic and phenotypic diversity essential for effective local transmission and survival (57). Reassortment in orbivirus occurs freely for all segments, whether between the eastern and western topotypes or among different serotypes (17, 57, 58). Further investigation is needed to evaluate how reassortment affects virus-host interactions, including virus fitness, host virulence, vector competence, and transmissibility, compared with parental viruses.

The SZ23C107 strain in Group D was identified as EHDV-5 by the phylogenetic analysis of its VP2 nt sequence with other EHDV-5 strains (Fig. 3), and they shared nt and aa identities of 92.5%–92.8%/94.4%–95.9% for Seg-2/VP2 and 92.6%–93.7%/98.6%–99.2% for Seg-6/VP5(Tables S5 and S6). Interestingly, the nt and aa sequences of Seg-2 (VP2), Seg-5 (VP6), and Seg-7 (VP7) of the MS23C056 strain presented the highest identity to the Japanese or Australian EHDV-5 strains. By contrast, the other segments presented the highest identity with the EHDV-1, -7, -8, and -10 strains isolated from China, Japan, or Australia (Table 3). The same results were observed in the phylogenetic tree constructed from the ORFs of all the segments (Fig. 2 and 3; Supplementary Figures). According to the phylogenetic trees for Seg-2, Seg-6, and Seg-7, the SZ23C107 strain is most closely related to EHDV-5 strains isolated from Japan and Australia. Furthermore, in the other phylogenetic tree, the SZ23C107 strain clustered with EHDV strains (serotypes 1, 7, 8, and 10) from China, Japan, and Australia. At present, only the complete genome sequences of the Australian EHDV-5 strain (AUS1977/01 CSIRO 157) (9) and the Japanese EHDV-5 strain (ON-11/E/16) (28) are available. The present study provides sequence data for a third EHDV-5 strain, which has the closest relationship and is grouped with EHDV strains from China, Japan, or Australia. Our results suggest that the EHDV-5 strain (SZ23C107) may share the most recent common ancestor with EHDV-5 strains from Japan and Australia; however, SZ23C107 has undergone long-term independent evolution within the Chinese region. Our findings again confirm the viewpoint Shirafuji et al. proposed that different EHDV serotypes have been continuously circulating in the Asia-Pacific region, causing genetic recombination and possibly evolving uniquely within specific areas (11, 28).

The OIE (2021) recommended using a serogroup-specific C-ELISA kit to detect antibodies against EHDV VP7 as the best option for monitoring EHDV infection (5). In this study, we first applied the C-ELISA method we previously developed (23) to detect bovine serum collected from 10 regions in Yunnan Province. The positive rate of EHDV antibodies ranged from 29.22% to 58.86% (*μ* = 43.09%), which aligns with our EHDV serological survey results in Yunnan Province from 2014 to 2019, where seroprevalence rates exceeded 30% (23). These findings indicate that the EHDV epidemic and its circulation continue to occur in Yunnan Province and that EHDV infection in cattle is widespread in this region. Menglian, Jinghong, Menghai, and Mengla Counties, located south of the Tropic of Cancer, had the highest positive EHDV antibodies (45.95%-58.86%). These four regions feature a tropical climate marked by consistently high temperatures, plentiful rainfall, and elevated humidity levels throughout the year. The remaining six regions have subtropical climates, with slightly lower EHDV positivity rates ranging from 29.33% to 44.24%. This finding aligns with our earlier observation that as latitude decreases, the EHDV infection rate increases from northern to southwestern China, rising from 0 to over 50% (23, 25). EHD is regarded as a livestock infectious disease transmitted by *Culicoides* spp., so this result is also consistent with the higher population density and vitality of *Culicoides* spp. in warmer and wetter weather regions (59, 60).

SNT results indicated that, except for EHDV-4, the seven other serotypes of EHDV (EHDV-1, -2, -5 to -8, and -10) are circulating in Yunnan Province, each with varying prevalence patterns. Except for Shizong, EHDV-6 and -7 presented the highest seroprevalence rates (Fig. 4). In Shizong, the positive rate of EHDV-1 was the highest; however, in other areas, EHDV-1 ranked third, following only EHDV-6 and -7. These findings indicate that the primary EHDV serotypes found in Yunnan are EHDV-1, -6, and -7, which aligns with the observation that multiple strains of EHDV-1 and -7 have been isolated from vectors or cattle across various regions of Yunnan (26, 56). The positivity rates for EHDV-2 and -8 were the lowest among the 10 studied areas. To date, there have been no reports of the isolation of EHDV-2 and -8 strains in China; however, antibodies against EHDV-8 have been detected in serum samples from Guangdong (25). Considering the strong pathogenicity of EHDV-2 and -8, surveillance of these two serotypes should be strengthened. In addition, the positivity rates for EHDV-5 and -10 fall between the highest and lowest rates. In 2013, EHDV-10 was first isolated from *Culicoides* (*C. tainanus*) collected in Yunnan, and over the next 5 years, antibodies against the virus were detected in the serum of local livestock (24). Currently, 5 years later, we continue to detect antibodies against EHDV-10 in cattle serum from Yunnan. This indicates the potential long-term prevalence of EHDV-10 in the region. Currently, EHDV-5 seroprevalence has only been reported in northern Guangdong, and there have been no confirmed cases of isolation of this serotype strain (25). This study not only isolated the EHDV-5 strain but also confirmed that EHDV-5 infection is prevalent in local cattle herds, indicating that the virus circulates in the local natural environment. However, the relationship between EHDV-5 from infected cattle in Guangdong and the EHDV-5 strain isolated in this study requires further investigation.

### Conclusions

In this study, we isolated four serotypes of EHDV strains (EHDV-1, -7, -5, and -6) from three *Culicoides* species (*C. tainanus*, *C. oxystoma*, and *C. jacobsoni*) collected in Yunnan Province, China. Furthermore, the three species of *Culicoides* spp. were found in relatively high proportions at three different collection sites, highlighting their potentially significant role in the local natural circulation of EHDV. We also found that the EHDV isolated in this study has the closest genetic relationship with the EHDV prevalent in Japan and Australia, suggesting that EHDVs circulating in the Asia-Pacific region may share the same gene pool. Remarkably, this is the first time the EHDV-5 strain has been isolated in China, following its initial isolation in Australia in 1977 and a second instance in Japan in 2016. Furthermore, findings from the sero-epidemiological investigation indicate that EHDV is present not only in natural vectors within the border areas of China, Laos, Myanmar, and Vietnam but also in local domestic cattle. However, the SNT epidemiology investigation revealed the presence of seven serotypes of EHDVs circulating in cattle in these regions, resulting in diverse prevalence rates and spatial distributions. The data obtained herein will aid in monitoring the evolutionary characteristics of EHDV and its potential to cause outbreaks in China and neighboring countries, enhancing our understanding of its genomic diversity and geographic distribution. Given these findings, improving the detection and monitoring of animal diseases caused by EHDV is increasingly essential.

## Data Availability

The nucleotide sequences of the complete genome sequences of JH24C022, JH24C130, MS23C056, and SZ23C107 strains obtained in this study were submitted to the GenBank database under accession numbers PV476106–PV476115, PV476116–PV476125, PV476126–PV476135, and PV476136–PV476145, respectively.

## SUPPLEMENTAL MATERIAL

Figures S1 to S5 msphere.00274-25-s0001.pdf Phylogenetic analysis based on the coding sequences of Seg-1, Seg-4, Seg-5, Seg-8, and Seg-9 of the four isolates in the study, with reference strains of recognized EHDV.

Table S1 msphere.00274-25-s0002.xlsx Information of all virus strains used in phylogenetic analysis.

Table S2 msphere.00274-25-s0003.docx Primers and probes for EHDV serotype-specific RT-qPCR assays.

Table S3 msphere.00274-25-s0004.docx Lengths of dsRNA segments 1-10, encoded putative proteins, and 5' and 3′ NCRs of the four EHDV strains.

Table S4 msphere.00274-25-s0005.xlsx Matrix of Seg-3/VP3 pairwise identity percentages of nucleotides and amino acids between different strains of EHDV.

Table S5 msphere.00274-25-s0006.xlsx Matrix of Seg-2/VP2 pairwise identity percentages of nucleotides and amino acids between different strains of EHDV.

Table S6 msphere.00274-25-s0007.xlsx Matrix of Seg-6/VP5 pairwise identity percentages of nucleotides and amino acids between different strains of EHDV.

Table S7 msphere.00274-25-s0008.docx Serotyping of EHDV in tested cattle sera collected from Yunnan.

ASM does not own the copyrights to Supplemental Material that may be linked to, or accessed through, an article. The authors have granted ASM a non-exclusive, world-wide license to publish the Supplemental Material files. Please contact the corresponding author directly for reuse.

## References

[1] Matthijnssens J, Attoui H, Bányai K, Brussaard CPD, Danthi P, del Vas M, Dermody TS, Duncan R, Fāng (方勤) Q, Johne R, Mertens PPC, Mohd Jaafar F, Patton JT, Sasaya (笹谷孝英) T, Suzuki (鈴木信弘) N, Wei (魏太云) T. 2022. ICTV Virus Taxonomy profile: Sedoreoviridae 2022. J Gen Virol 103:10. doi:10.1099/jgv.0.001782 PMC12643109 36215107

[2] Maclachlan NJ, Zientara S, Savini G, Daniels PW. 2015. Epizootic haemorrhagic disease. Rev Sci Tech 34:341–351. doi:10.20506/rst.34.2.2361 26601439

[3] Savini G, Afonso A, Mellor P, Aradaib I, Yadin H, Sanaa M, Wilson W, Monaco F, Domingo M. 2011. Epizootic heamorragic disease. Res Vet Sci 91:1–17. doi:10.1016/j.rvsc.2011.05.004 21665237

[4] Shope RE, Macnamara LG, Mangold R. 1960. A virus-induced epizootic hemorrhagic disease of the virginia white-tailed deer (odocoileus virginianus). J Exp Med 111:155–170. doi:10.1084/jem.111.2.155 19867168 PMC2137250

[5] OIE. Diseases, infections and infestations listed by the OIE. Available from: https://www.woah.org/fileadmin/Home/eng/Health_standards/tahc/current/chapitre_oie_listed_disease.pdf. Retrieved 08 Oct 2022.

[6] Jiménez-Cabello L, Utrilla-Trigo S, Lorenzo G, Ortego J, Calvo-Pinilla E. 2023. Epizootic hemorrhagic disease virus: current knowledge and emerging perspectives. Microorganisms 11:1339. doi:10.3390/microorganisms11051339 37317313 PMC10224379

[7] Anthony SJ, Maan N, Maan S, Sutton G, Attoui H, Mertens PPC. 2009. Genetic and phylogenetic analysis of the core proteins VP1, VP3, VP4, VP6 and VP7 of epizootic haemorrhagic disease virus (EHDV). Virus Res 145:187–199. doi:10.1016/j.virusres.2009.07.011 19632280

[8] Belhouchet M, Mohd Jaafar F, Firth AE, Grimes JM, Mertens PPC, Attoui H. 2011. Detection of a fourth orbivirus non-structural protein. PLoS One 6:e25697. doi:10.1371/journal.pone.0025697 22022432 PMC3192121

[9] Anthony SJ, Maan S, Maan N, Kgosana L, Bachanek-Bankowska K, Batten C, Darpel KE, Sutton G, Attoui H, Mertens PPC. 2009. Genetic and phylogenetic analysis of the outer-coat proteins VP2 and VP5 of epizootic haemorrhagic disease virus (EHDV): comparison of genetic and serological data to characterise the EHDV serogroup. Virus Res 145:200–210. doi:10.1016/j.virusres.2009.07.012 19632281

[10] Bréard E, Viarouge C, Donnet F, Sailleau C, Rossi S, Pourquier P, Vitour D, Comtet L, Zientara S. 2020. Evaluation of a commercial ELISA for detection of epizootic haemorrhagic disease antibodies in domestic and wild ruminant sera. Transbound Emerg Dis 67:2475–2481. doi:10.1111/tbed.13586 32310339

[11] Shirafuji H, Kato T, Yamakawa M, Tanaka T, Minemori Y, Yanase T. 2017. Characterization of genome segments 2, 3 and 6 of epizootic hemorrhagic disease virus strains isolated in Japan in 1985-2013: identification of their serotypes and geographical genetic types. Infect Genet Evol 53:38–46. doi:10.1016/j.meegid.2017.05.010 28506840

[12] Yang H, Li Z, Wang J, Li Z, Yang Z, Liao D, Zhu J, Li H. 2020. Novel serotype of epizootic hemorrhagic disease virus, China. Emerg Infect Dis 26:3081–3083. doi:10.3201/eid2612.191301 33219797 PMC7706924

[13] Omori T, Inaba Y, Morimoto T, Tanaka Y, Ishitani R. 1969. Ibaraki virus, an agent of epizootic disease of cattle resembling bluetongue. I. Epidemiologic, clinical and pathologic observations and experimental transmission to calves. Jpn J Microbiol 13:139–157. doi:10.1111/j.1348-0421.1969.tb00447.x 4309396

[14] Uchinuno Y, Ito T, Goto Y, Miura Y, Ishibashi K, Itou T, Sakai T. 2003. Differences in Ibaraki virus RNA segment 3 sequences from three epidemics. J Vet Med Sci 65:1257–1263. doi:10.1292/jvms.65.1257 14665759

[15] Garrett EF, Po E, Bichi ER, Hexum SK, Melcher R, Hubner AM. 2015. Clinical disease associated with epizootic hemorrhagic disease virus in cattle in Illinois. J Am Vet Med Assoc 247:190–195. doi:10.2460/javma.247.2.190 26133219

[16] Bréard E, Sailleau C, Hamblin C, Graham SD, Gourreau JM, Zientara S. 2004. Outbreak of epizootic haemorrhagic disease on the island of Réunion. Vet Rec 155:422–423. doi:10.1136/vr.155.14.422 15508843

[17] Rodrigues TCS, Viadanna PHO, Subramaniam K, Hawkins IK, Jeon AB, Loeb JC, Krauer JMC, Lednicky JA, Wisely SM, Waltzek TB. 2022. Characterization of a novel reassortant epizootic hemorrhagic disease virus serotype 6 strain isolated from diseased white-tailed deer (Odocoileus virginianus) on a Florida farm. Viruses 14:1012. doi:10.3390/v14051012 35632753 PMC9146129

[18] Yadin H, Brenner J, Bumbrov V, Oved Z, Stram Y, Klement E, Perl S, Anthony S, Maan S, Batten C, Mertens PPC. 2008. Epizootic haemorrhagic disease virus type 7 infection in cattle in Israel. Vet Rec 162:53–56. doi:10.1136/vr.162.2.53 18192658

[19] Kedmi M, Van Straten M, Ezra E, Galon N, Klement E. 2010. Assessment of the productivity effects associated with epizootic hemorrhagic disease in dairy herds. J Dairy Sci 93:2486–2495. doi:10.3168/jds.2009-2850 20494156

[20] Ben Hassine T, García-Carrasco JM, Sghaier S, Thabet S, Lorusso A, Savini G, Hammami S. 2024. Epidemiological analyses of the first incursion of the epizootic hemorrhagic disease virus serotype 8 in Tunisia, 2021-2022. Viruses 16:362. doi:10.3390/v16030362 38543728 PMC10974811

[21] Ruiz-Fons F, García-Bocanegra I, Valero M, Cuadrado-Matías R, Relimpio D, Martínez R, Baz-Flores S, Gonzálvez M, Cano-Terriza D, Ortiz JA, Gortázar C, Risalde MA. 2024. Emergence of epizootic hemorrhagic disease in red deer (Cervus elaphus), Spain, 2022. Vet Microbiol 292:110069. doi:10.1016/j.vetmic.2024.110069 38569324

[22] Lorusso A, Cappai S, Loi F, Pinna L, Ruiu A, Puggioni G, Guercio A, Purpari G, Vicari D, Sghaier S, Zientara S, Spedicato M, Hammami S, Ben Hassine T, Portanti O, Breard E, Sailleu C, Ancora M, Di Sabatino D, Morelli D, Calistri P, Savini G. 2023. Epizootic hemorrhagic disease virus serotype 8, Italy, 2022. Emerg Infect Dis 29:1063–1065. doi:10.3201/eid2905.221773 37081599 PMC10124640

[23] Duan Y, Yang Z, Zhu P, Xiao L, Li Z, Li Z, Li L, Zhu J. 2022. A serologic investigation of epizootic hemorrhagic disease virus in China between 2014 and 2019. Virol Sin 37:513–520. doi:10.1016/j.virs.2022.06.005 35718300 PMC9437609

[24] He Y, Meng J, Li N, Li Z, Wang D, Kou M, Yang Z, Li Y, Zhang L, Wang J. 2024. Isolation of Epizootic Hemorrhagic Disease Virus serotype 10 from Culicoides tainanus and associated infections in Livestock in Yunnan, China. Viruses 16:175. doi:10.3390/v16020175 38399951 PMC10892452

[25] Lv MN, Zhu JB, Liao SQ, Yang ZX, Lin XH, Qi NS, Chen QL, Wu CY, Li J, Cai HM, Zhang JF, Hu JJ, Xiao WW, Zhang X, Sun MF. 2023. Seroprevalence of epizootic hemorrhagic disease virus in Guangdong cattle farms during 2013–2017, China. Viruses 15:1263. doi:10.3390/v15061263 37376563 PMC10303234

[26] Duan Y, Yang Z, Bellis G, Xie J, Li L. 2022. Full genome sequencing of three Sedoreoviridae viruses isolated from Culicoides spp. (Diptera, Ceratopogonidae) in China. Viruses 14:971. doi:10.3390/v14050971 35632713 PMC9145729

[27] Zhen-xing Y, Mei-ling K, Zhan-hong L, De-fang L, Lei X, Jian-bo Z, Lin C, Hua-chun L, Heng Y. 2019. The first isolation and identification of serotype 6 epizootic haemorrhagic disease virus in Yunnan province of China. Chin J Preventive Vet Med 41:1113–1119. doi:10.3969/j.issn.1008-0589.201903014

[28] Shirafuji H, Murota K, Kishida N, Suda Y, Yanase T. 2023. Complete genome sequences of epizootic hemorrhagic disease virus serotypes 5 and 6 isolated in Japan. Arch Virol 168:230. doi:10.1007/s00705-023-05853-z 37578645

[29] Wang J, Liu G. 2023. Hematophagous midges of Yunnan Province (Diptera: Ceratopogonidae), p 68–265. China Agriculture Press, Beijing, China.

[30] Yang Z, Li N, He Y, Meng J, Wang J. 2023. Genetic characterization of DH13M98, Umatilla virus, isolated from Culex tritaeniorhynchus Giles in Yunnan Province, China. Vector Borne Zoonotic Dis 23:35–43. doi:10.1089/vbz.2022.0031 36595376

[31] Attoui H, Billoir F, Cantaloube JF, Biagini P, de Micco P, de Lamballerie X. 2000. Strategies for the sequence determination of viral dsRNA genomes. J Virol Methods 89:147–158. doi:10.1016/s0166-0934(00)00212-3 10996648

[32] Hofmann M, Griot C, Chaignat V, Perler L, Thür B. 2008. Blauzungenkrankheit erreicht die Schweiz. Schweiz Arch Tierheilkd 150:49–56. doi:10.1024/0036-7281.150.2.49 18369049

[33] Maan S, Rao S, Maan NS, Anthony SJ, Attoui H, Samuel AR, Mertens PPC. 2007. Rapid cDNA synthesis and sequencing techniques for the genetic study of bluetongue and other dsRNA viruses. J Virol Methods 143:132–139. doi:10.1016/j.jviromet.2007.02.016 17433453

[34] Potgieter AC, Page NA, Liebenberg J, Wright IM, Landt O, van Dijk AA. 2009. Improved strategies for sequence-independent amplification and sequencing of viral double-stranded RNA genomes. J Gen Virol 90:1423–1432. doi:10.1099/vir.0.009381-0 19264638

[35] Jackman SD, Vandervalk BP, Mohamadi H, Chu J, Yeo S, Hammond SA, Jahesh G, Khan H, Coombe L, Warren RL, Birol I. 2017. ABySS 2.0: resource-efficient assembly of large genomes using a Bloom filter. Genome Res 27:768–777. doi:10.1101/gr.214346.116 28232478 PMC5411771

[36] Luo R, Liu B, Xie Y, Li Z, Huang W, Yuan J, He G, Chen Y, Pan Q, Liu Y, et al.. 2012. SOAPdenovo2: an empirically improved memory-efficient short-read de novo assembler. Gigascience 1:18. doi:10.1186/2047-217X-1-18 23587118 PMC3626529

[37] Katoh K, Asimenos G, Toh H. 2009. Multiple alignment of DNA sequences with MAFFT. Methods Mol Biol 537:39–64. doi:10.1007/978-1-59745-251-9_3 19378139

[38] Zhou ZJ, Qiu Y, Pu Y, Huang X, Ge XY. 2020. BioAider: an efficient tool for viral genome analysis and its application in tracing SARS-CoV-2 transmission. Sustain Cities Soc 63:102466. doi:10.1016/j.scs.2020.102466 32904401 PMC7455202

[39] Worwa G, Chaignat V, Feldmann J, Thür B. 2013. Detection of neutralizing antibodies against bluetongue virus serotype 8 by an optimized plasma neutralization test. J Virol Methods 188:168–174. doi:10.1016/j.jviromet.2012.08.027 23000751

[40] Meng J, Wang F, He Y, Li N, Yang Z, Yao J, Wang S, Xiong G, Yuan Z, Xia H, Wang J. 2023. In vivo and in vitro characterization of a new Oya virus isolate from Culicoides spp. and its seroprevalence in domestic animals in Yunnan, China. PLoS Negl Trop Dis 17:e0011374. doi:10.1371/journal.pntd.0011374 37319258 PMC10306208

[41] Zhenxing Y, Zhanhong L, Ziang S, Jianbo Z, Zhuoran L, Huachun L, Jiarui X, Defang L, Heng Y. 2020. Establishment and preliminary application of a serotype-specific fluorescence quantitative RT-PCR method for epidemic hemorrhagic disease virus (EHDV). Chin J Virol 36:897–906. doi:10.13242/j.cnki.bingduxuebao.003750

[42] Wilson WC, Ruder MG, Klement E, Jasperson DC, Yadin H, Stallknecht DE, Mead DG, Howerth E. 2015. Genetic characterization of epizootic hemorrhagic disease virus strains isolated from cattle in Israel. J Gen Virol 96:1400–1410. doi:10.1099/vir.0.000089 25701817

[43] Mellor PS, Boorman J, Baylis M. 2000. Culicoides biting midges: their role as arbovirus vectors. Annu Rev Entomol 45:307–340. doi:10.1146/annurev.ento.45.1.307 10761580

[44] Borkent A, Dominiak P. 2020. Catalog of the biting midges of the world (Diptera: Ceratopogonidae). Zootaxa 4787:zootaxa.4787.1.1. doi:10.11646/zootaxa.4787.1.1 33056456

[45] Duan YL, Yang ZX, Bellis G, Li L. 2021. Isolation of Tibet Orbivirus from Culicoides jacobsoni (Diptera, Ceratopogonidae) in China. Parasit Vectors 14:432. doi:10.1186/s13071-021-04899-9 34454575 PMC8401062

[46] Li N, Meng J, He Y, Wang W, Wang J. 2023. Potential roles of Culicoides spp. (Culicoides imicola, Culicoides oxystoma) as biological vectors of bluetongue virus in Yuanyang of Yunnan, P. R. China. Front Cell Infect Microbiol 13:1283216. doi:10.3389/fcimb.2023.1283216 38274733 PMC10809989

[47] Yang Z, He Y, Chen Y, Meng J, Li N, Li S, Wang J. 2023. Full genome characterization and evolutionary analysis of Banna virus isolated from Culicoides, mosquitoes and ticks in Yunnan, China. Front Cell Infect Microbiol 13:1283580. doi:10.3389/fcimb.2023.1283580 38035340 PMC10687475

[48] Wang J, Li H, He Y, Zhou Y, Xin A, Liao D, Meng J. 2017. Isolation of Tibet orbivirus from Culicoides and associated infections in livestock in Yunnan, China. Virol J 14:105. doi:10.1186/s12985-017-0774-9 28595631 PMC5488374

[49] Meng J, He Y, Li N, Yang Z, Fu S, Wang D, Xin A, Wang J, Liang G. 2024. Akabane virus isolated from biting midges and its infection in local domestic animal, Yunnan, China: a field and laboratory investigation. Front Cell Infect Microbiol 14:1434045. doi:10.3389/fcimb.2024.1434045 39897479 PMC11783144

[50] Duan YL, Li L, Bellis G, Yang ZX, Li HC. 2021. Detection of bluetongue virus in Culicoides spp. in southern Yunnan Province, China. Parasit Vectors 14:68. doi:10.1186/s13071-020-04518-z 33482882 PMC7821528

[51] Kato T, Shirafuji H, Tanaka S, Sato M, Yamakawa M, Tsuda T, Yanase T. 2016. Bovine arboviruses in Culicoides biting midges and sentinel cattle in Southern Japan from 2003 to 2013. Transbound Emerg Dis 63:e160–e172. doi:10.1111/tbed.12324 25597441

[52] Ruder MG, Stallknecht DE, Allison AB, Mead DG, Carter DL, Howerth EW. 2016. Host and potential vector susceptibility to an emerging orbivirus in the United States: epizootic hemorrhagic disease virus serotype 6. Vet Pathol 53:574–584. doi:10.1177/0300985815610387 26459518

[53] Noronha LE, Cohnstaedt LW, Richt JA, Wilson WC. 2021. Perspectives on the changing landscape of epizootic hemorrhagic disease virus control. Viruses 13:2268. doi:10.3390/v13112268 34835074 PMC8618044

[54] Qin S, Yang H, Zhang Y, Li Z, Lin J, Gao L, Liao D, Cao Y, Ren P, Li H, Wu J. 2018. Full genome sequence of the first bluetongue virus serotype 21 (BTV-21) isolated from China: evidence for genetic reassortment between BTV-21 and bluetongue virus serotype 16 (BTV-16). Arch Virol 163:1379–1382. doi:10.1007/s00705-018-3718-9 29392498

[55] Yang H, Xiao L, Meng J, Xiong H, Gao L, Liao D, Li H. 2016. Complete genome sequence of a Chuzan virus strain isolated for the first time in mainland China. Arch Virol 161:1073–1077. doi:10.1007/s00705-015-2734-2 26733292

[56] Qi Y, Wang F, Chang J, Zhang Y, Zhu J, Li H, Yu L. 2019. Identification and complete-genome phylogenetic analysis of an epizootic hemorrhagic disease virus serotype 7 strain isolated in China. Arch Virol 164:3121–3126. doi:10.1007/s00705-019-04412-9 31538253

[57] Allison AB, Goekjian VH, Potgieter AC, Wilson WC, Johnson DJ, Mertens PPC, Stallknecht DE. 2010. Detection of a novel reassortant epizootic hemorrhagic disease virus (EHDV) in the USA containing RNA segments derived from both exotic (EHDV-6) and endemic (EHDV-2) serotypes. J Gen Virol 91:430–439. doi:10.1099/vir.0.015651-0 19828758

[58] Ren N, Wang X, Liang M, Tian S, Ochieng C, Zhao L, Huang D, Xia Q, Yuan Z, Xia H. 2021. Characterization of a novel reassortment Tibet orbivirus isolated from Culicoides spp. in Yunnan, PR China. J Gen Virol 102:001645. doi:10.1099/jgv.0.001645 34494948 PMC8567429

[59] Purse BV, Carpenter S, Venter GJ, Bellis G, Mullens BA. 2015. Bionomics of temperate and tropical Culicoides midges: knowledge gaps and consequences for transmission of Culicoides-borne viruses. Annu Rev Entomol 60:373–392. doi:10.1146/annurev-ento-010814-020614 25386725

[60] Hudson AR, McGregor BL, Shults P, England M, Silbernagel C, Mayo C, Carpenter M, Sherman TJ, Cohnstaedt LW. 2023. Culicoides-borne Orbivirus epidemiology in a changing climate. J Med Entomol 60:1221–1229. doi:10.1093/jme/tjad098 37862060

