# Supplementary material for: Isolation of four serotypes of epizootic hemorrhagic disease virus from Culicoides spp. and their associated infections in cattle in Yunnan, China
Source: mSphere. 2025 Jul 31;10(8):e00274-25. doi: 10.1128/msphere.00274-25 (PMC12379597; doi:10.1128/msphere.00274-25)
Supplement: Figures S1 to S5 — Phylogenetic analysis based on the coding sequences of Seg-1, Seg-4, Seg-5, Seg-8, and Seg-9 of the four isolates in the study, with reference strains of recognized EHDV. [file msphere.00274-25-s0001.pdf]

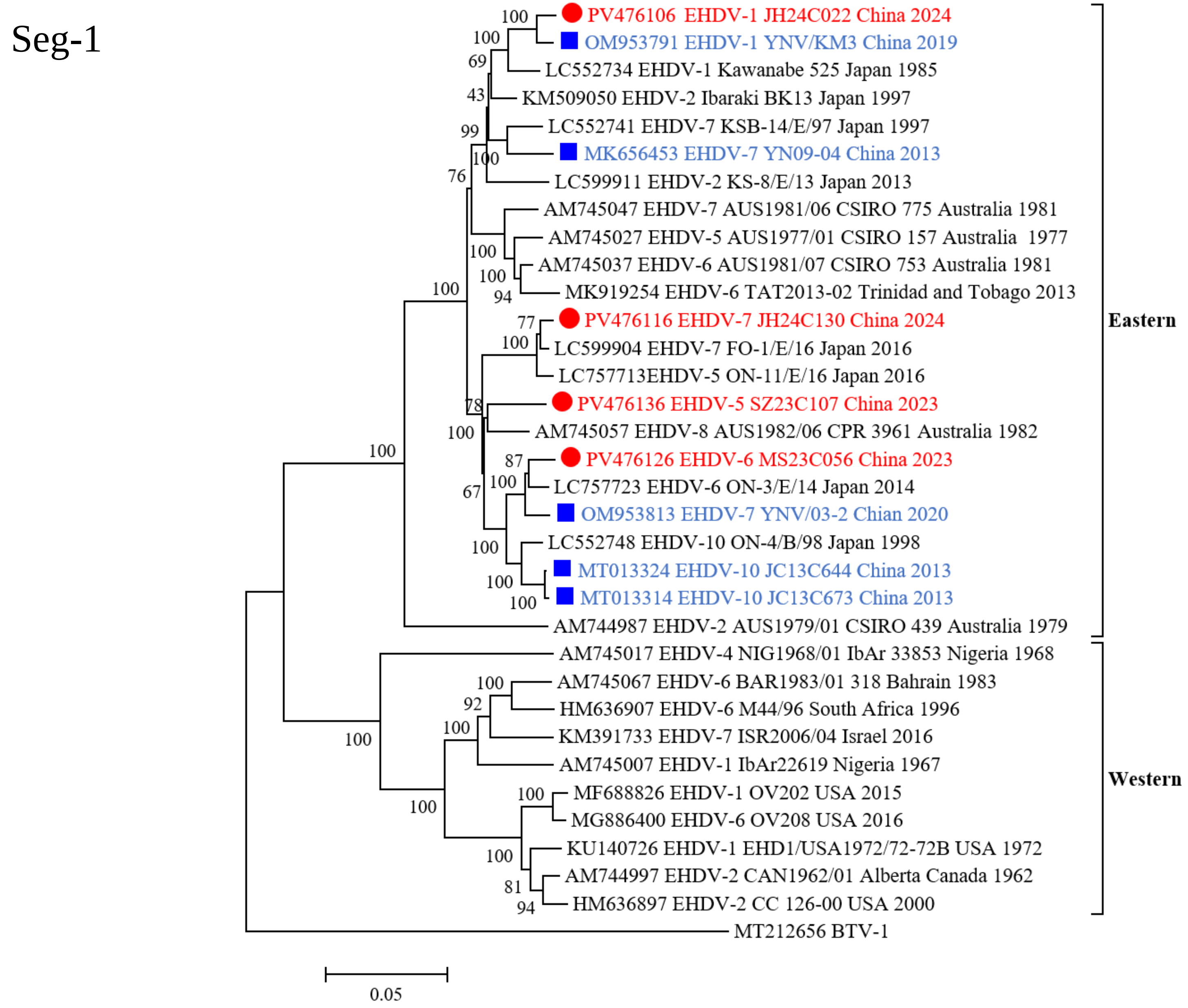

Supplementary Figure S1. Phylogenetic analysis based on the coding sequences of Seg-1 of the four isolates in the study with reference strains of recognized EHDV.

Each reference EHDV strain is denoted as 'GenBank accession number_ Serotype_ Strains number_ Country_ Date'. Outgroup viruses are denoted as 'GenBank accession number_ Virus name'. Red dots represent the isolates in this study, while blue squares denote other EHDV strains isolated in China.

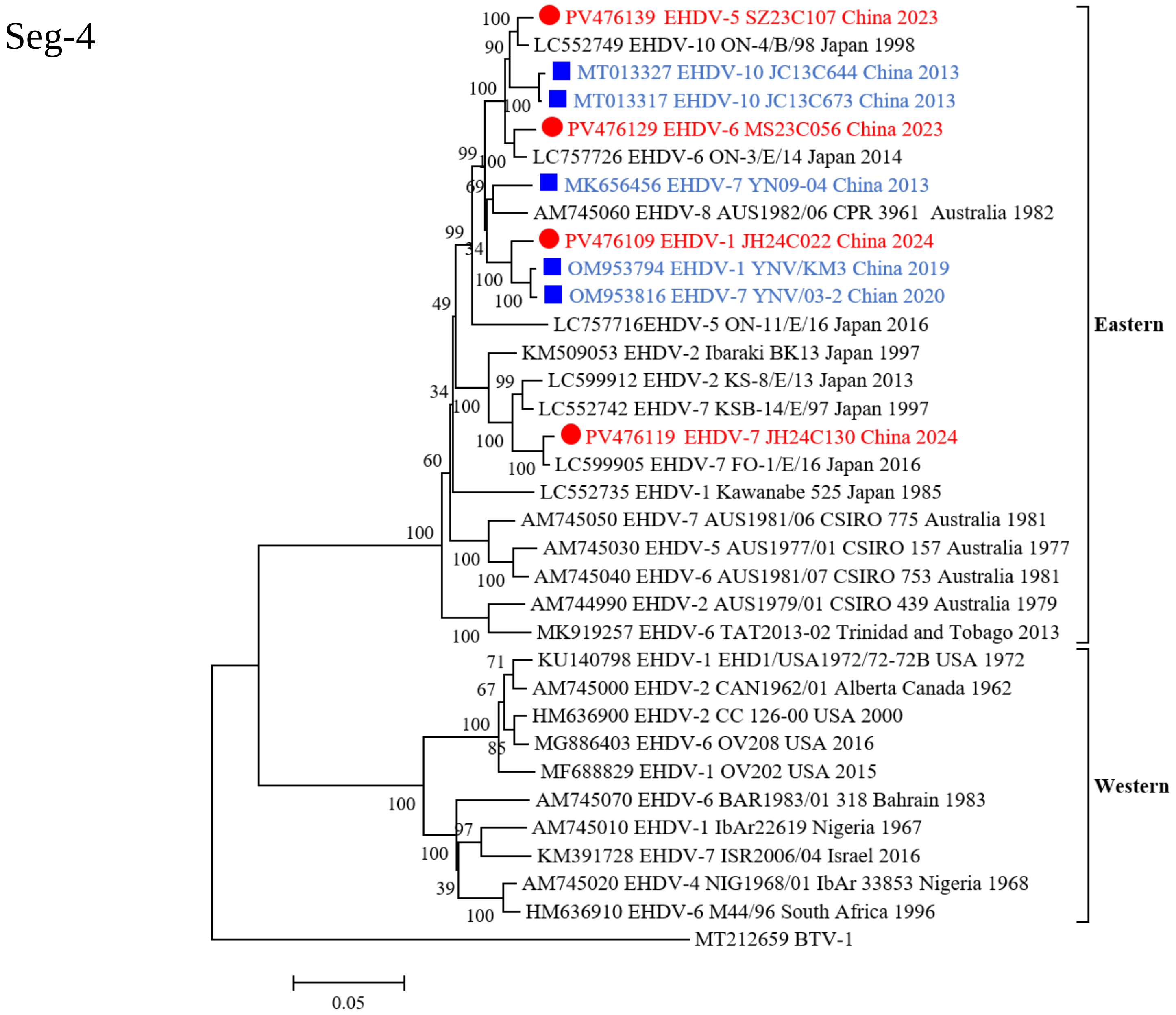

Supplementary Figure S2. Phylogenetic analysis based on the coding sequences of Seg-4 of the four isolates in the study with reference strains of recognized EHDV.

Each reference EHDV strain is denoted as 'GenBank accession number_ Serotype_ Strains number_ Country_ Date'. Outgroup viruses are denoted as 'GenBank accession number_ Virus name'. Red dots represent the isolates in this study, while blue squares denote other EHDV strains isolated in China.

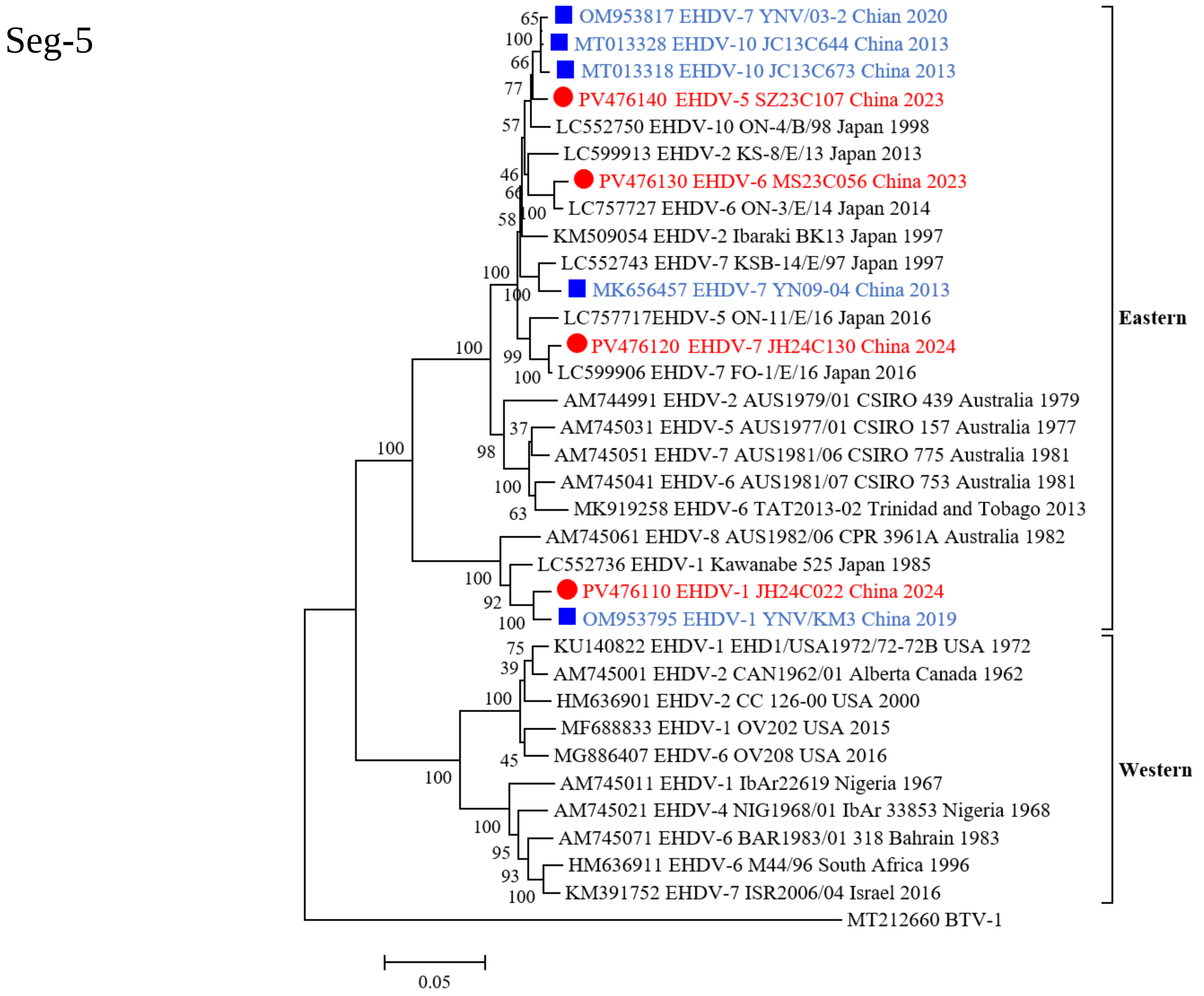

Supplementary Figure S3. Phylogenetic analysis based on the coding sequences of Seg-5 of the four isolates in the study with reference strains of recognized EHDV.

Each reference EHDV strain is denoted as 'GenBank accession number_ Serotype_ Strains number_ Country_ Date'. Outgroup viruses are denoted as 'GenBank accession number_ Virus name'. Red dots represent the isolates in this study, while blue squares denote other EHDV strains isolated in China.

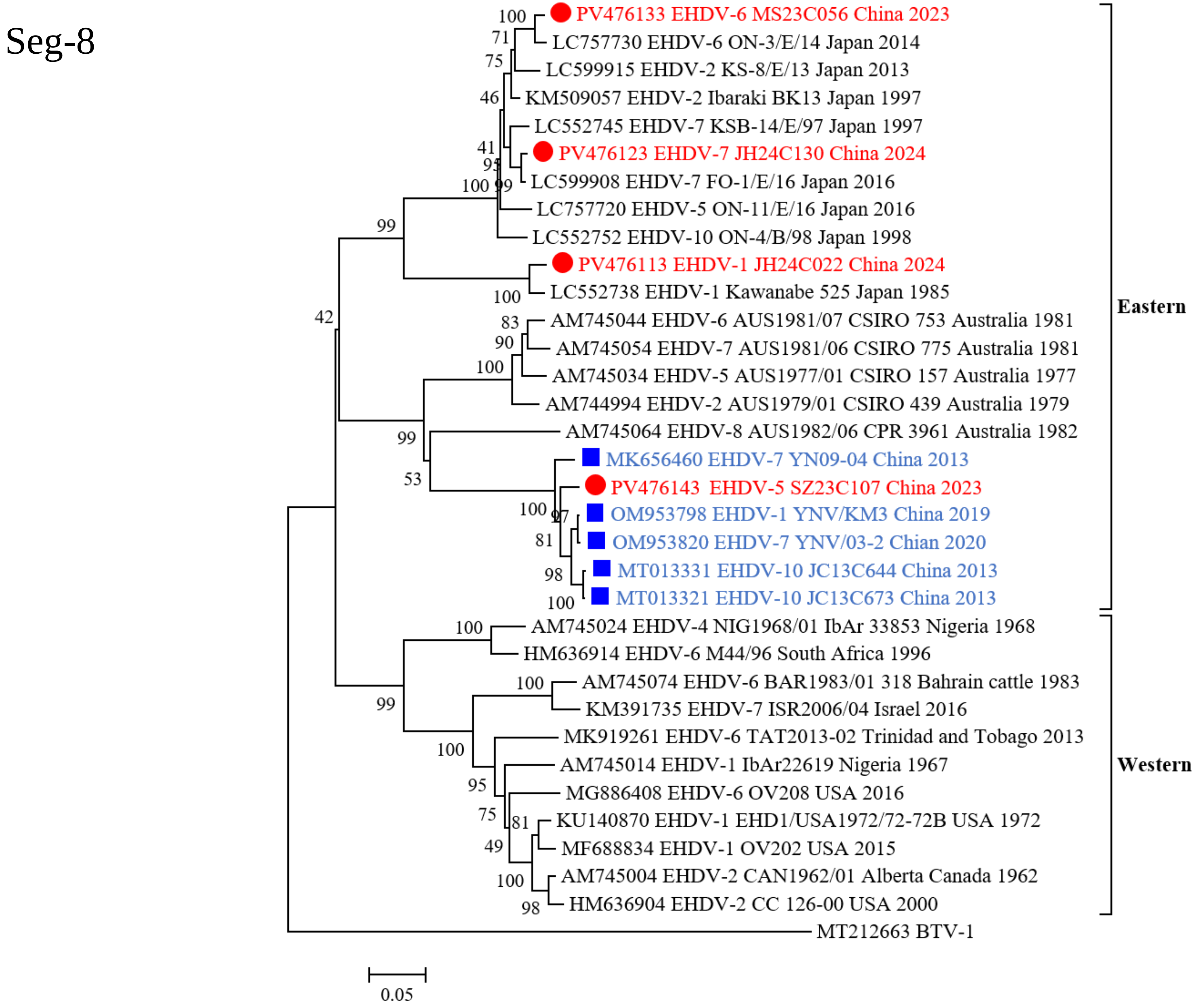

Supplementary Figure S4. Phylogenetic analysis based on the coding sequences of Seg-8 of the four isolates in the study with reference strains of recognized EHDV.

Each reference EHDV strain is denoted as 'GenBank accession number_ Serotype_ Strains number_ Country_ Date'. Outgroup viruses are denoted as 'GenBank accession number_ Virus name'. Red dots represent the isolates in this study, while blue squares denote other EHDV strains isolated in China.

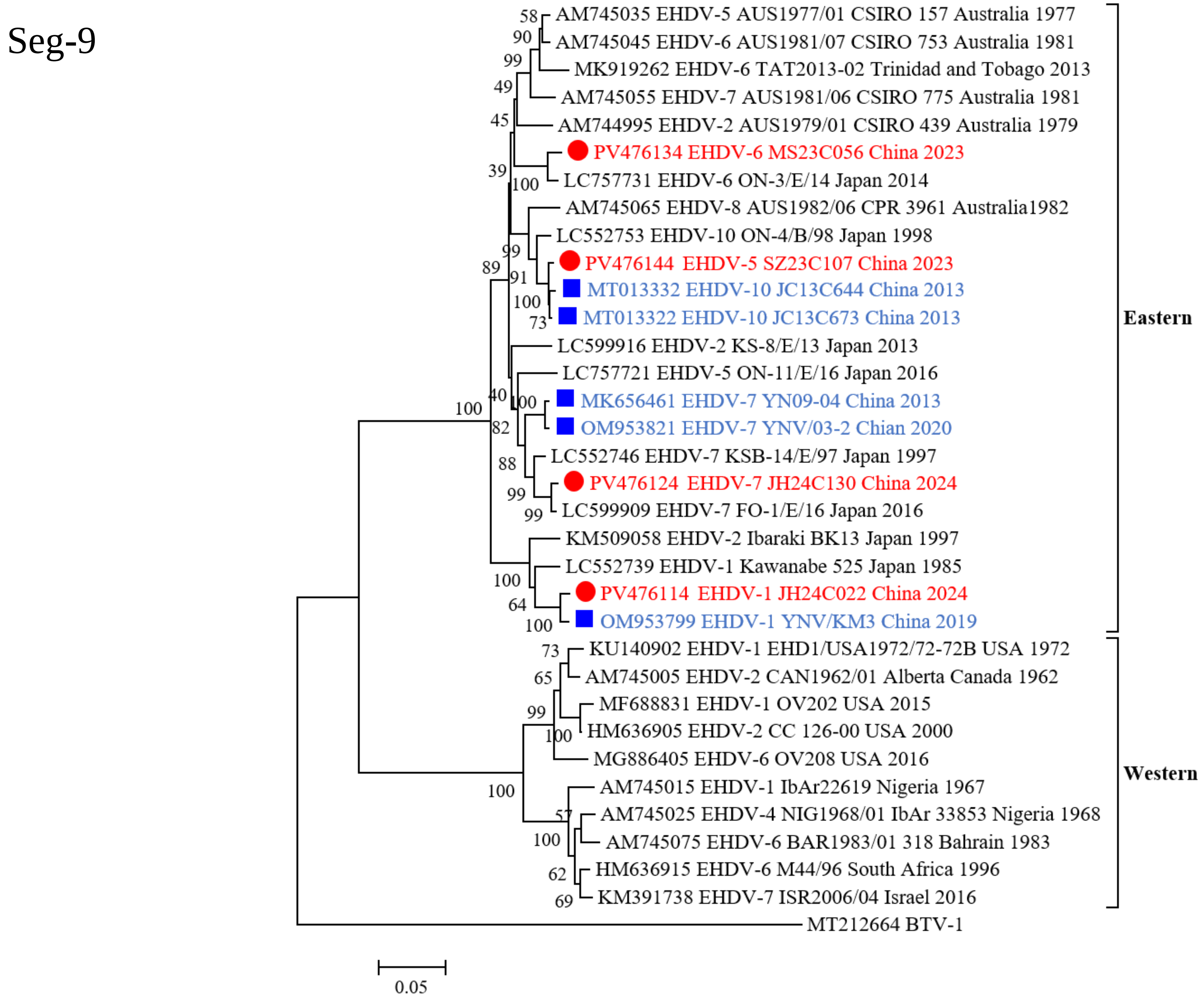

Supplementary Figure S5. Phylogenetic analysis based on the coding sequences of Seg-9 of the four isolates in the study with reference strains of recognized EHDV.

Each reference EHDV strain is denoted as 'GenBank accession number_ Serotype_ Strains number_ Country_ Date'. Outgroup viruses are denoted as 'GenBank accession number_ Virus name'. Red dots represent the isolates in this study, while blue squares denote other EHDV strains isolated in China.
