# Supplementary material for: Isolation of four serotypes of epizootic hemorrhagic disease virus from Culicoides spp. and their associated infections in cattle in Yunnan, China
Source: mSphere. 2025 Jul 31;10(8):e00274-25. doi: 10.1128/msphere.00274-25 (PMC12379597; doi:10.1128/msphere.00274-25)
Supplement: Table S2 — Primers and probes for EHDV serotype-specific RT-qPCR assays. [file msphere.00274-25-s0003.docx]

Table S2 Primers and probes for EHDV serotype-specific RT-qPCR assays.

| Serotypes | Names of primers and probes | Sequence (5'→3') | Location | Amplicon length (bp) |
| --- | --- | --- | --- | --- |
| EHDV-1 | EHDV1-Probe | CGTATCYAATCGGTTCACTTCCATCC | 2520-2546 | 71 |
|  | EHDV1-YG-F | GCTGTGTTTGATTATTGGATC | 2498-2519 |  |
|  | EHDV1-YG-R | GCACGAGTTCTCAATCTATC | 2529-2549 |  |
| EHDV-2 | EHDV2-Probe | AGCAACTCTCTTCTTTCAACTCCAACCG | 681-775 | 94 |
|  | EHDV2-YG-F | TTACCACGAGCGTTAATCCTG | 721-742 |  |
|  | EHDV2-YG-R | TCCTAAACCTTCCAACAGCG | 881-901 |  |
| EHDV-4 | EHDV4-Probe | CTTGTCTTCCCAAAGTTACCCCGCAT | 2810-2836 | 145 |
|  | EHDV4-YG-F | TTGTGAAACTTGCTGACTTGC | 2738-2759 |  |
|  | EHDV4-YG-R | AGTTGCCTTCCCTGTATATTCTC | 2837-2860 |  |
| EHDV-5 | EHDV5-Probe | CACTTCCGATGTATTCCTCTATGCGAC | 2008-2035 | 107 |
|  | EHDV5-YG-F | CCCTAARGAAGAGGATGAAAG | 1954-1976 |  |
|  | EHDV5-YG-R | TGCTCAGTTGTATACTTGTATTC | 2015-2038 |  |
| EHDV-6 | EHDV6-Probe | ATCCACTTTCTCGTCACTTTGTCTCTGC | 867-895 | 104 |
|  | EHDV6-YG-F | CCGCTGATAACACGATTAGAT | 827-848 |  |
|  | EHDV6-YG-R | TGCTTTCCCTTYGCCTCTAAG | 889-910 |  |
| EHDV-7 | EHDV7-Probe | CCGGACGTAATTGCCARGCCATATCT | 1199-1225 | 203 |
|  | EHDV7-YG-F | AACGATAGGTTTGAGTTAGTGAG | 1102-1125 |  |
|  | EHDV7-YG-R | TCGTGTCTCCTAYTTCCCTATT | 1259-1282 |  |
| EHDV-8 | EHDV8-Probe | TCACATACTCTACGGCATCCATCAACG | 1239-1266 | 108 |
|  | EHDV8-YG-F | GGGTGTGTCTACTATGGTTTAC | 1184-1205 |  |
|  | EHDV8-YG-R | ACGGGTCATAAATGTTTGTCG | 1250-1271 |  |
| EHDV-10 | EHDV10-Probe | AGCAACTCTCTTCTTTCAACTCCAACCG | 721-742 | 200 |
|  | EHDV10-YG-F | TTACCACGAGCGTTAATCCTG | 881-901 |  |
|  | EHDV10-YG-R | TCCTAAACCTTCCAACAGCG | 783-811 |  |
