# Supplementary material for: Isolation of four serotypes of epizootic hemorrhagic disease virus from Culicoides spp. and their associated infections in cattle in Yunnan, China
Source: mSphere. 2025 Jul 31;10(8):e00274-25. doi: 10.1128/msphere.00274-25 (PMC12379597; doi:10.1128/msphere.00274-25)
Supplement: Table S3 — Lengths of dsRNA segments 1-10, encoded putative proteins, and 5' and 3′ NCRs of the four EHDV strains. [file msphere.00274-25-s0004.docx]

Table S3 Lengths of dsRNA segments 1–10, encoded putative proteins, 5' and 3′ NCRs of the four EHDV strains.

| Strains | Segment | Protein encoded | Segment length (bp) | Size of protein (aa) | Protein molecular mass (kDa) | G+C content (%) | 5' NCR (bp) | Terminal sequence (5'–3') | 3' NCR (bp) | Stop-codon | GenBank Accession no. |
| --- | --- | --- | --- | --- | --- | --- | --- | --- | --- | --- | --- |
| JH24C022 | Seg-1 | VP1 (Pol) | 3942 | 1302 | 149.77 | 40.13 | 11 | **GUUAAA**AT---ACA**CUUAC** | 22 | TAG | PV476106 |
|  | Seg-2 | VP2 (OC1) | 2968 | 971 | 113.05 | 40.09 | 16 | **GUUAAA**TT---CCA**CUUAC** | 36 | TGA | PV476107 |
|  | Seg-3 | VP3 (T2) | 2768 | 899 | 103.05 | 41.8 | 17 | **GUUAAA**TT---ACA**CUUAC** | 51 | TGA | PV476108 |
|  | Seg-4 | VP4 (Cap) | 1984 | 644 | 76.11 | 41.23 | 8 | **GUUAAA**AC---CAC**CUUAC** | 41 | TAG | PV476109 |
|  | Seg-5 | NS1 (Tup) | 1769 | 551 | 64.57 | 43.7 | 31 | **GUUAAA**AA---ACA**CUUAC** | 82 | TGA | PV476110 |
|  | Seg-6 | VP5 (OC2) | 1640 | 528 | 59.40 | 42.93 | 24 | **GUUAAA**AA---ACA**CUUAC** | 29 | TGA | PV476111 |
|  | Seg-7 | VP7 (T13) | 1162 | 349 | 38.25 | 43.8 | 17 | **GUUAAA**AT---CAA**CUUAC** | 95 | TGA | PV476112 |
|  | Seg-8 | NS2 (Vip) | 1186 | 373 | 43.16 | 42.75 | 19 | **GUUAAA**AA---ACA**CUUAC** | 45 | TAA | PV476113 |
|  | Seg-9 | VP6 (Hel) | 1071 | 336 | 37.31 | 45.28 | 14 | **GUUAAA**AA---AAA**CUUAC** | 46 | TAA | PV476114 |
|  | Seg-10 | NS3 (VRP) | 810 | 228 | 25.49 | 45.31 | 20 | **GUUAAA**AA---ACA**CUUAC** | 103 | TAG | PV476115 |
|  | Total |  | 19300 | 6181 | 710.16 | 41.93 | 177 | **GUUAAA**--------------**CUUAC** | 550 |  |  |
| JH24C130 | Seg-1 | VP1 (Pol) | 3942 | 1302 | 149.69 | 39.8 | 11 | **GUUAAA**AT---ACA**CUUAC** | 22 | TAG | PV476116 |
|  | Seg-2 | VP2 (OC1) | 3002 | 982 | 114.85 | 39.74 | 17 | **GUUAAA**TT---AGT**CUUAC** | 36 | TAG | PV476117 |
|  | Seg-3 | VP3 (T2) | 2768 | 899 | 103.03 | 42.23 | 17 | **GUUAAA**TT---ACA**CUUAC** | 51 | TGA | PV476118 |
|  | Seg-4 | VP4 (Cap) | 1984 | 644 | 76.02 | 42.74 | 8 | **GUUAAA**AC---CAC**CUUAC** | 41 | TAG | PV476119 |
|  | Seg-5 | NS1 (Tup) | 1769 | 551 | 64.55 | 43.19 | 31 | **GUUAAA**AA---ACA**CUUAC** | 82 | TGA | PV476120 |
|  | Seg-6 | VP5 (OC2) | 1641 | 527 | 58.97 | 44.12 | 27 | **GUUAAA**AA---CCA**CUUAC** | 30 | TGA | PV476121 |
|  | Seg-7 | VP7 (T13) | 1162 | 349 | 38.10 | 42.43 | 17 | **GUUAAA**AT---CAA**CUUAC** | 95 | TGA | PV476122 |
|  | Seg-8 | NS2 (Vip) | 1186 | 373 | 43.38 | 42.33 | 19 | **GUUAAA**AA---ACA**CUUAC** | 45 | TAA | PV476123 |
|  | Seg-9 | VP6 (Hel) | 1074 | 337 | 37.52 | 46.93 | 14 | **GUUAAA**AA---AAA**CUUAC** | 46 | TAA | PV476124 |
|  | Seg-10 | NS3 (VRP) | 810 | 228 | 25.48 | 44.94 | 20 | **GUUAAA**AA---ACA**CUUAC** | 103 | TAG | PV476125 |
|  | Total |  | 19338 | 6192 | 711.60 | 42.04 | 181 | **GUUAAA**--------------**CUUAC** | 551 |  |  |
| Strains | Segment | Protein encoded | Segment length (bp) | Size of protein (aa) | Protein molecular mass (kDa) | G+C content (%) | 5' NCR (bp) | Terminal sequence (5'–3') | 3' NCR (bp) | Stop-codon | GenBank Accession no. |
| MS23C056 | Seg-1 | VP1 (Pol) | 3942 | 1302 | 149.75 | 40.13 | 11 | **GUUAAA**AT---ACA**CUUAC** | 22 | TAG | PV476126 |
|  | Seg-2 | VP2 (OC1) | 2971 | 972 | 112.75 | 40.26 | 16 | **GUUAAA**TT---ATA**CUUAC** | 36 | TGA | PV476127 |
|  | Seg-3 | VP3 (T2) | 2768 | 899 | 103.06 | 41.98 | 17 | **GUUAAA**TT---ACA**CUUAC** | 51 | TAA | PV476128 |
|  | Seg-4 | VP4 (Cap) | 1984 | 644 | 76.09 | 41.18 | 8 | **GUUAAA**AC---CAC**CUUAC** | 41 | TAG | PV476129 |
|  | Seg-5 | NS1 (Tup) | 1769 | 551 | 64.50 | 43.7 | 31 | **GUUAAA**AA---ACA**CUUAC** | 82 | TGA | PV476130 |
|  | Seg-6 | VP5 (OC2) | 1642 | 527 | 59.06 | 43.73 | 28 | **GUUAAA**AA---CAG**CUUAC** | 30 | TGA | PV476131 |
|  | Seg-7 | VP7 (T13) | 1162 | 349 | 38.08 | 43.72 | 17 | **GUUAAA**AT---CAA**CUUAC** | 95 | TGA | PV476132 |
|  | Seg-8 | NS2 (Vip) | 1186 | 373 | 43.38 | 42.66 | 19 | **GUUAAA**AA---ACA**CUUAC** | 45 | TAA | PV476133 |
|  | Seg-9 | VP6 (Hel) | 1073 | 332 | 36.89 | 46.23 | 14 | **GUUAAA**AA---AAA**CUUAC** | 60 | TAA | PV476134 |
|  | Seg-10 | NS3 (VRP) | 810 | 228 | 25.62 | 43.95 | 20 | **GUUAAA**AA---ACA**CUUAC** | 103 | TAG | PV476135 |
|  | Total |  | 19307 | 6177 | 709.18 | 42.03 | 181 | **GUUAAA**--------------**CUUAC** | 565 |  |  |
| SZ23C107 | Seg-1 | VP1 (Pol) | 3942 | 1302 | 149.80 | 40.18 | 11 | **GUUAAA**AT---ACA**CUUAC** | 22 | TAG | PV476136 |
|  | Seg-2 | VP2 (OC1) | 3019 | 987 | 114.48 | 40.81 | 18 | **GUUAAA**TT---ACA**CUUAC** | 37 | TAG | PV476137 |
|  | Seg-3 | VP3 (T2) | 2768 | 899 | 103.00 | 42.81 | 17 | **GUUAAA**TT---ACA**CUUAC** | 51 | TAG | PV476138 |
|  | Seg-4 | VP4 (Cap) | 1984 | 644 | 76.16 | 41.33 | 8 | **GUUAAA**AC---CAC**CUUAC** | 41 | TAG | PV476139 |
|  | Seg-5 | NS1 (Tup) | 1769 | 551 | 64.53 | 42.74 | 31 | **GUUAAA**AA---ACA**CUUAC** | 82 | TGA | PV476140 |
|  | Seg-6 | VP5 (OC2) | 1644 | 529 | 59.26 | 45.68 | 25 | **GUUAAA**AG---ACA**CUUAC** | 29 | TGA | PV476141 |
|  | Seg-7 | VP7 (T13) | 1162 | 349 | 38.21 | 43.03 | 17 | **GUUAAA**AT---CAA**CUUAC** | 95 | TGA | PV476142 |
|  | Seg-8 | NS2 (Vip) | 1192 | 375 | 43.67 | 45.89 | 19 | **GUUAAA**AA---ACA**CUUAC** | 45 | TAA | PV476143 |
|  | Seg-9 | VP6 (Hel) | 1074 | 337 | 37.52 | 44.79 | 14 | **GUUAAA**AA---AAA**CUUAC** | 46 | TAA | PV476144 |
|  | Seg-10 | NS3 (VRP) | 810 | 228 | 25.56 | 44.32 | 20 | **GUUAAA**AA---ACA**CUUAC** | 103 | TAG | PV476145 |
|  | Total |  | 19364 | 6201 | 712.19 | 42.42 | 180 | **GUUAAA**--------------**CUUAC** | 551 |  |  |

Conserved nucleotide sequences in 5′- and 3′-terminals are shown in bold. RNA-dependent RNA Polymerase (Pol), Outer capsid protein (OC1), Major subcore protein (T2), Minor core protein-Capping enzyme (CaP), Tubule protein (TuP), Outer capsid protein (OC2), Major core-surface protein (T13), Viral inclusion body protein (ViP), Minor core protein-helicase enzyme (Hel), Virus release protein (VRP).
