# Supplementary material for: Isolation of four serotypes of epizootic hemorrhagic disease virus from Culicoides spp. and their associated infections in cattle in Yunnan, China
Source: mSphere. 2025 Jul 31;10(8):e00274-25. doi: 10.1128/msphere.00274-25 (PMC12379597; doi:10.1128/msphere.00274-25)
Supplement: Table S7 — Serotyping of EHDV in tested cattle sera collected from Yunnan. [file msphere.00274-25-s0008.docx]

Table S7 Serotyping of EHDV in tested cattle sera (*n* = 1,000) collected from Yunnan.

|  | EHDV-1 | EHDV-2 | EHDV-5 | EHDV-6 | EHDV-7 | EHDV-8 | EHDV-10 |
| --- | --- | --- | --- | --- | --- | --- | --- |
| Number of positive sera | 208 | 60 | 110 | 321 | 300 | 42 | 91 |
| Ratio (%) | 20.8 | 6.0 | 11.0 | 32.1 | 30.0 | 4.2 | 9.1 |
| 95% CI of ratios | 18.28-23.32 | 4.53-7.47 | 9.06-12.94 | 29.21-34.99 | 27.16-32.84 | 2.96-5.44 | 7.32-10.88 |
